# Preparing for the tiny predators: Community readiness and perceptions of copepod-based dengue control in eastern guatemala

**DOI:** 10.1371/journal.pntd.0014654

**Published:** 2026-09-03

**Authors:** Valérie R. Louis, Carlos Alberto Montenegro-Quinoñez, Till Bärnighausen, Peter Dambach

**Affiliations:** 1 Heidelberg Institute of Global Health (HIGH), Heidelberg University Hospital, Heidelberg, Germany; 2 Instituto de Investigaciones, Centro Universitario de Zacapa, Universidad de San Carlos de Guatemala, Guatemala, Guatemala; Instituto Oswaldo Cruz, BRAZIL

## Abstract

**Background:**

Dengue remains a persistent public health challenge in eastern Guatemala, particularly in communities where irregular water supply, domestic water storage, and limited sanitation infrastructure create favorable conditions for *Aedes aegypti* proliferation. As concerns increase about the sustainability of chemical vector control, biological approaches such as the use of predatory copepods are receiving renewed attention. However, successful implementation depends not only on biological efficacy, but also on community awareness, acceptance, and willingness to adopt unfamiliar control methods.

**Methodology/Principal Findings:**

We conducted a cross-sectional Knowledge, Attitudes, and Practices (KAP) survey among 346 adult household representatives in eight urban and rural communities in the departments of Zacapa and Chiquimula, Guatemala. The study assessed dengue-related knowledge, household prevention practices, perceived risk, and attitudes toward potential future copepod-based biological control. Most respondents correctly identified mosquitoes as the vector of dengue, but knowledge of breeding sites and preventive measures was less comprehensive and varied by educational level. Preventive practices were inconsistent: 37% of households reported taking no action to eliminate mosquito breeding sites, and only a minority reported using multiple prevention strategies. Although prior familiarity with copepods was limited, willingness to consider their use was high when linked to clear information, technical support, and assurances regarding water safety. Concerns focused primarily on the introduction of organisms into stored water and lack of knowledge about the method, particularly in urban communities.

**Conclusions/Significance:**

The findings indicate a substantial gap between basic dengue awareness and routine household prevention practices in eastern Guatemala. At the same time, the high conditional openness toward copepod-based control suggests that biological approaches may be socially acceptable if introduced through trusted local actors, transparent communication, and community engagement. These results support the further exploration of copepod-based interventions as a complementary, non-chemical vector control strategy. However, community acceptance should not be interpreted as evidence of effectiveness; rigorous field trials are needed to evaluate entomological impact, operational feasibility, safety, and sustainability under local environmental and household water-storage conditions.

## Author summary

Dengue fever continues to pose a major public health challenge in Guatemala, particularly in the eastern departments of Zacapa and Chiquimula, where household water storage and limited sanitation infrastructure create favorable breeding conditions for Aedes aegypti mosquitoes. As resistance to chemical insecticides increases, there is growing interest in alternative and more sustainable mosquito control methods. One promising biological approach involves the use of tiny aquatic crustaceans called copepods, which prey on mosquito larvae in water containers.

To better understand whether such an approach could be accepted in local communities, we conducted interviews with 346 residents across eight urban and rural communities in eastern Guatemala. We explored what people know about dengue, how they try to prevent mosquitoes, and how they perceive the possible future use of copepods for mosquito control. While most participants correctly identified mosquitoes as the source of dengue transmission, many households reported limited preventive practices. At the same time, respondents expressed considerable openness toward copepod-based control if sufficient information, technical guidance, and reassurance regarding water safety were provided.

These findings suggest that communities may be receptive to innovative biological vector control strategies when interventions are accompanied by trusted communication and local engagement. However, acceptance alone does not demonstrate effectiveness. Further field-based studies are needed to evaluate whether copepod-based approaches can sustainably reduce mosquito populations and dengue risk under real-world conditions in Guatemala.

## Introduction

Dengue fever, transmitted primarily by *Aedes aegypti* mosquitoes, poses a growing public health challenge in tropical and subtropical regions worldwide. In Guatemala, the burden of dengue has intensified over recent years, particularly in the northeastern departments of Zacapa and Chiquimula. Rapid urbanization, rising temperatures, seasonal rainfall, irregular water supply, and limited sanitation infrastructure have created ideal conditions for mosquito proliferation. Despite continued efforts by the Ministry of Health, dengue remains a leading cause of morbidity, with recurrent outbreaks placing considerable strain on health systems [1–4]. Recent surveillance and epidemiological analyses indicate that dengue outbreaks in Guatemala have increased in both frequency and intensity over recent decades, particularly in environmentally and socioeconomically vulnerable regions characterized by rapid urbanization, irregular water access, and infrastructural limitations [2]. Conventional vector control in Guatemala relies heavily on chemical measures such as space spraying and the use of larvicides. However, growing insecticide resistance in *Aedes* populations, along with concerns over the long-term sustainability and environmental impact of these methods, has spurred interest in complementary, non-chemical approaches [5,6]. In this context, biological control strategies offer promising alternatives, particularly those that can be integrated into community-based programs [7].

One such approach involves the use of copepods, small aquatic crustaceans, that can act as predators of mosquito larvae in water storage containers. Copepod-based control has demonstrated remarkable success in Vietnam, where extensive pilot programs in both urban and rural settings have shown that copepods can sustainably suppress *Aedes* populations when maintained by the community. Studies from northern and central Vietnam have documented not only high larval mortality but also long-term reductions in dengue incidence, especially when copepods were deployed alongside health education and community mobilization efforts [8,9]. These programs highlight that the success of copepod-based interventions depends not only on biological performance, but also on community acceptance, local maintenance, trust in the intervention, and sustained behavioral engagement, factors commonly explored through Knowledge, Attitudes, and Practices (KAP) approaches in dengue control research. In Latin America, however, experience with copepod-based vector control remains limited. The Guatemalan context presents a number of potential advantages and challenges. On the one hand, high reliance on water storage, both in rural and peri-urban households, provides ample opportunity for copepod use in domestic containers [10]. On the other hand, local perceptions around water quality, unfamiliar organisms, and the visibility of mosquito control efforts may shape community acceptance of biological methods. Understanding these perceptions is especially important as dengue outbreaks increase in frequency and severity, and the need grows for integrated vector management strategies that are locally sustainable and socially acceptable.

To address these knowledge gaps, we conducted a cross-sectional Knowledge, Attitudes, and Practices (KAP) study in eight communities across northeastern Guatemala. The study aimed to assess awareness of dengue transmission and prevention practices, perceptions of dengue risk, and community attitudes toward the potential future use of copepods as a biological vector control strategy. Particular attention was paid to perceived barriers, facilitators, trust, and information needs related to this unfamiliar intervention approach.

By generating community-level insights into both existing prevention practices and the social acceptability of copepod-based control, this study seeks to inform the development of locally appropriate, community-based vector control strategies in Guatemala and similar settings. However, the study does not evaluate entomological effectiveness or operational impact, which will require future field-based implementation research.

## Methods

### Ethics and consent

Ethical approval for the study was obtained from the Postgraduate Bioethics Committee of the University of San Carlos of Guatemala (Ref. EEPVirtual.165.2021). The study was conducted in accordance with national ethical guidelines and the principles of the Declaration of Helsinki.

Prior to participation, all respondents received an explanation of the study objectives, procedures, voluntary nature of participation, and data handling procedures in Spanish. Written informed consent was obtained electronically from all participants before the interview was initiated.

To protect participant confidentiality, no directly identifying personal information was included in the analytical dataset. Survey responses were stored in password-protected electronic databases accessible only to authorized study personnel. Data were analyzed in anonymized form, and results are presented only in aggregated format to prevent identification of individual participants or households.

### Design

The study adopted a cross-sectional Knowledge, Attitudes, and Practices (KAP) design to assess dengue-related knowledge, perceptions, preventive behaviors, and attitudes toward potential future biological vector control measures in eight communities located in the departments of Zacapa and Chiquimula, Guatemala (Pueblo Modelo and El Bordo in Zacapa; Sabana Grande, San Esteban, Santa Elena, Vado Hondo, Petapilla and El Jurgallon in Chiquimula). The study was primarily designed to provide descriptive and exploratory insights into community readiness and perceptions regarding copepod-based dengue vector control under local household and environmental conditions.

### Sample size

The sample size was initially calculated to estimate the prevalence of key knowledge, attitude, and practice indicators related to dengue prevention and biological vector control with a 95% confidence level and a 5% margin of error. Assuming a conservative expected proportion of 50%, which yields the maximum sample size for prevalence estimation, a minimum sample size of 384 respondents was calculated under assumptions of simple random sampling.

However, because the study was conducted across eight geographically distinct communities using a cluster-based household sampling approach, and due to logistical and fieldwork constraints, approximately 40–50 interviews per community were targeted, resulting in a final sample size target of approximately 340 respondents. No formal design effect adjustment was applied. The study was therefore intended primarily to generate descriptive and exploratory community-level insights rather than highly precise inferential estimates. With a final sample size of 346 respondents, the study retained an acceptable level of precision for exploratory analyses and prevalence estimation.

### Survey population

Participants aged 18 years and older who had resided in the selected communities for at least six months were eligible for inclusion. One adult representative per household was interviewed. Permission to enter household premises was requested before recruitment.

Households within communities were selected using a systematic random sampling approach. In each locality, field teams first selected a random starting point and subsequently approached every 10th to 20th household depending on housing density and settlement structure. Sampling routes were distributed across the entire community to ensure broad geographic coverage and reduce spatial clustering of interviews within individual neighborhoods or streets. If no eligible participant was available or consent was not granted, the next household was approached.

### Questionnaire and data collection

A structured questionnaire was developed using the KoBo Toolbox software (kobotoolbox.org), which operates under a Creative Commons Attribution-Non-Commercial license (CC BY-NC 4.0). Questionnaire development was informed by a review of published dengue-related KAP surveys and previously validated instruments used in comparable settings [11,12].

The questionnaire included sections on sociodemographic characteristics, dengue-related knowledge, perceptions of dengue risk, household prevention practices, water storage behavior, attitudes toward vector control, and perceptions regarding the potential future use of copepods for biological mosquito control. Perception- and attitude-related questions were assessed using predefined categorical response options.

The questionnaire was developed in English and translated into Spanish by bilingual members of the research team familiar with the local context and terminology. The translated version was reviewed collaboratively by Spanish-speaking researchers and field staff to ensure linguistic clarity, contextual appropriateness, and conceptual consistency.

Prior to the main survey, the questionnaire was pilot-tested in the community of La Laguna, Zacapa, to identify ambiguities, assess respondent comprehension, and refine wording and response categories where necessary.

Trained interviewers conducted face-to-face household interviews using tablet computers or mobile phones. Data collection covered multiple thematic domains, including dengue symptoms and transmission, mosquito breeding sites, preventive behaviors, information sources, attitudes toward vector control interventions, and willingness to consider future copepod-based biological control approaches (S2 Table).

### Analysis

Open-ended responses (e.g., “other” categories) were reviewed and thematically grouped into existing or newly created categories where appropriate. For six multiple-response questions, compound scores were constructed to summarize dengue-related knowledge, attitudes, and preventive practices (S2 Table). Each score was based on predefined expert-informed criteria. Correct responses received one point, while incorrect responses and “do not know” answers received zero points. Depending on the question, the maximum achievable score ranged from 1 to 11.

Descriptive statistics were summarized using frequencies and percentages for categorical variables and means with standard deviations for continuous variables. Missing data were generally infrequent and were handled using complete-case analysis for the respective variables, resulting in slightly varying denominators across analyses.

Statistical analyses were conducted using Stata/BE 17.0 for Windows (StataCorp LLC, College Station, TX, USA). Associations between categorical variables were initially explored using chi-square tests.

Because the score variables represented count data, Poisson regression models were used to assess associations between explanatory variables and score outcomes. Binary outcomes were analyzed using logistic regression models. Regression coefficients are presented as log-rate estimates for Poisson models and log-odds estimates for logistic models, together with their 95% confidence intervals.

Prior to fitting the final regression models, an exploratory stepwise variable selection procedure at the 0.1 significance level was used to identify candidate explanatory variables among the sociodemographic and household infrastructure indicators. Final multivariable models included variables retained after exploratory screening and considered epidemiologically relevant. The regression analyses should therefore be interpreted as exploratory rather than confirmatory.

To account for potential intra-community correlation resulting from the cluster-based sampling design, clustered standard errors were estimated at the community level using the Stata command “vce(cluster community)”. Model diagnostics did not indicate substantial overdispersion in the final Poisson regression models. Statistical significance was assessed at the 0.05 level.

## Results

A total of 357 questionnaires were collected across eight communities in the departments of Zacapa and Chiquimula. Of these, 346 respondents (97%) provided informed consent and were included in the final analysis. The survey aimed to assess community knowledge, attitudes, and practices (KAP) related to dengue prevention, as well as receptiveness to novel biological control measures such as copepods.

### Descriptive

#### Sample characteristics.

Demographic characteristics of the study population are shown in Table 1. The majority of respondents were female (77.8%, n = 266). The mean age of participants was 48.1 years (SD = 16.6), with no major differences between sexes. Approximately 18.5% of respondents reported no formal education, while 22.6% had completed education beyond the secondary level.

**Table 1:** Demographic information by sex (percentages are calculated within the women, men, and total columns for each variable).

Variables and Categories	Women		Men		Total	
	N	%	N	%	N	%
**Sex**	266	77.8	76	22.2	342	100
**Age: mean** ± **SD [range]**	47.2 ± 16.5 [18-84]		50.9 ± 16.8 [22-81]		48.1 ± 16.6 [18-84]	
**Education**	**N**	**%**	**N**	**%**	**N**	**%**
No formal education	49	18.5	14	18.5	63	18.5
Primary school	117	44.2	34	44.7	151	44.3
Secondary school	39	14.7	11	14.5	50	14.7
Above secondary school	60	22.6	17	22.4	77	22.6
**Total**	**265**	**100.0**	**76**	**100.0**	**341**	**100.0**
**Owner of place of residence**	**N**	**%**	**N**	**%**	**N**	**%**
No	44	16.5	9	11.8	53	15.5
Yes	222	83.5	67	88.2	289	84.5
Total	**266**	**100.0**	**76**	**100.0**	**342**	**100.0**
**Communities (N to S): Z = Zacapa, C = Chiquimula**	**N**	**%**	**N**	**%**	**N**	**%**
Pueblo Modelo, Z (Rural)	33	12.4	9	11.8	42	12.3
El Bordo, Z (Urban)	32	12.0	10	13.2	42	12.3
Petapilla, C (Urban)	32	12.0	8	10.5	40	11.7
El Jurgallon, C (Rural)	34	12.8	10	13.2	44	12.9
San Esteban, C (Urban)	38	14.3	7	9.2	45	13.2
Sabana Grande, C (Urban)	33	12.4	10	13.2	43	12.6
Vado Hondo, C (Urban)	29	10.9	12	15.8	41	12.0
Santa Elena, C (Urban)	35	13.2	10	13.2	45	13.2
**Total**	**266**	**100.0**	**76**	**100.0**	**342**	**100.0**

Denominators vary slightly across variables because of missing responses.

Home ownership was high, with 84.5% of participants reporting they owned the place where they lived. This indicator was consistent across both urban and rural communities. Community-level representation was balanced, with samples from each of the eight localities, which included both urban and rural settings (Table 1).

#### Household infrastructure and environmental risk factors.

**Fig 1:**
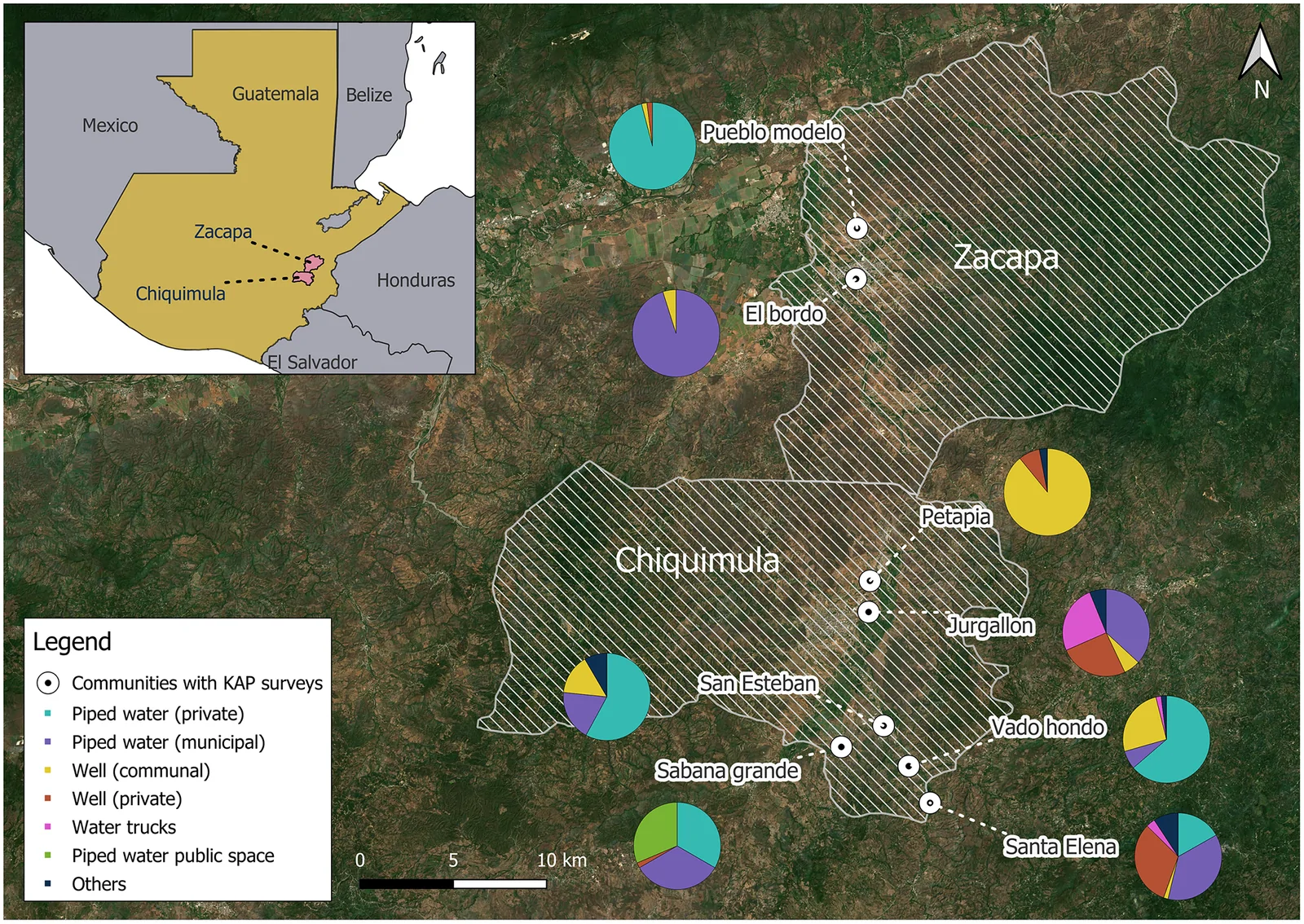
Map locating the eight communities in which the KAP survey was conducted. The pie diagrams show the type of water supply that is used in the interviewed households. Basemap shapefile obtained from United Nations Office for the Coordination of Humanitarian Affairs (OCHA), (https://data.humdata.org/dataset/cod-ab-gtm). Background imagery derived from Copernicus Sentinel-2 satellite data accessed through the Sentinel Hub EO Browser 2026 (https://apps.sentinel-hub.com/eo-browser/). Copernicus Sentinel data are provided on a free and open basis under the Copernicus data policy (https://cds.climate.copernicus.eu/licences/ec-sentinel). The figure was generated by the authors using openly accessible geographic data compatible with CC BY licensing requirements.

Fig 1 provides a geospatial overview of the surveyed communities, alongside pie charts depicting household water source types. Considerable variation was observed in water access. While some communities—such as Pueblo Modelo, El Bordo, Petapilla, and San Esteban— primarily relied on piped water into homes, others like Sabana Grande and Vado Hondo had a heterogeneous mix of water sources, including communal wells, public standpipes, and water truck deliveries.

Sanitation facilities also varied across sites. Septic tanks were the most common sanitation system in six of the eight localities, covering between 73% and 100% of households. However, only 20% or fewer households were connected to municipal sewage networks, except in Sabana Grande (65%) and Pueblo Modelo (38.1%). In rural areas, latrines and blind pits remained prevalent. Importantly, household infrastructure indicators, such as access to electricity (83–100%) and presence of an outdoor area (80–100%), were consistently high across communities.

Water storage practices posed a significant entomological risk. In all localities, a majority of households reported storing water, often in open containers. Only 25% of households used exclusively covered containers, leaving the vast majority vulnerable to *Aedes aegypti* breeding (S1 Table).

### Knowledge, attitude and practice scores

**Fig 2:**
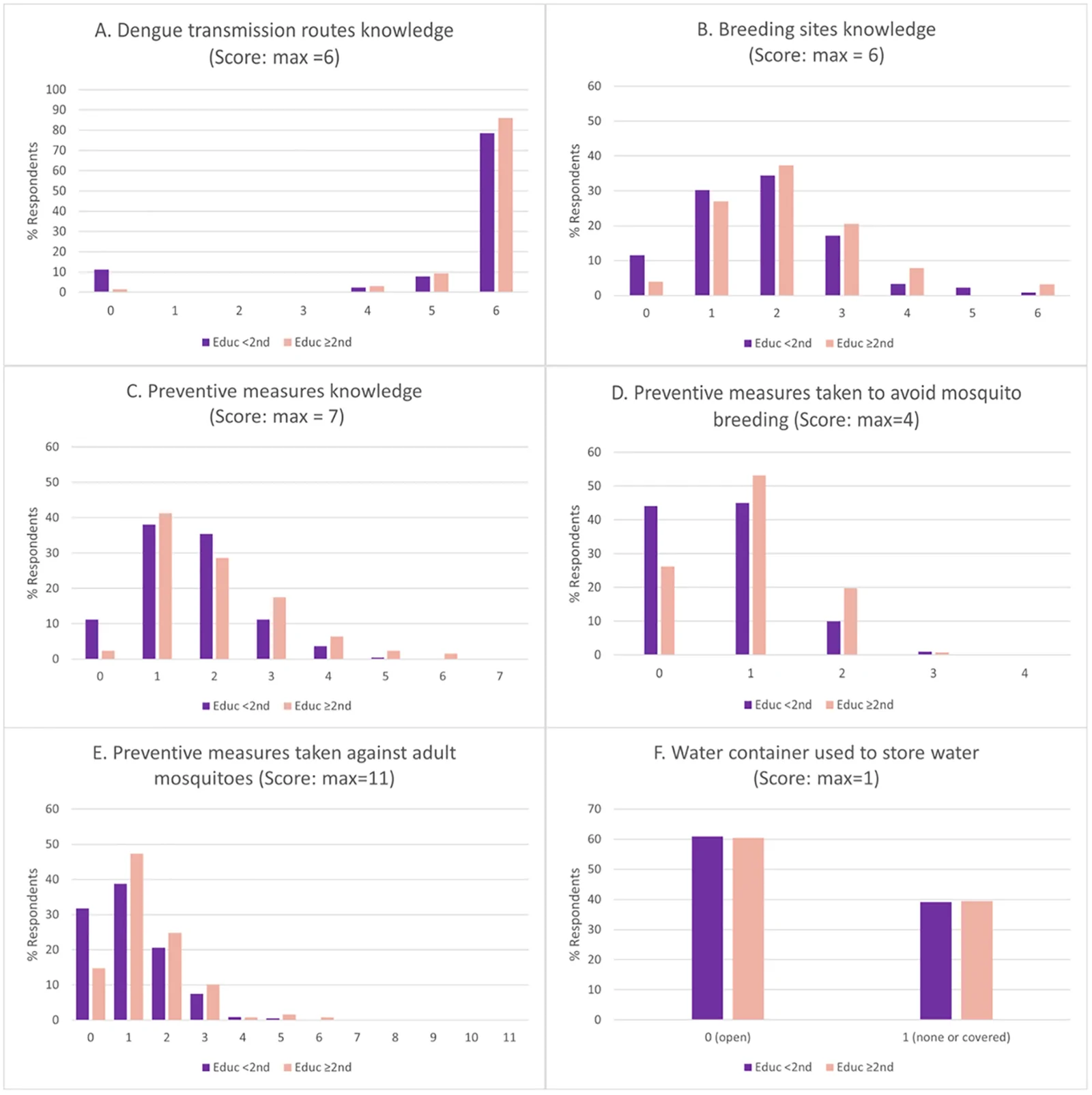
Scores in function of education level. (COCODES: *Consejo comunitario de desarrollo* = Community council for development). Knowledge: panels A-C; Practice: panels D-F.

KAP scores were computed for multiple indicators, capturing the breadth of respondent understanding and behavior related to dengue prevention. Sex and home ownership were not significantly associated with the analyzed score outcomes, whereas educational attainment showed consistent positive associations with several knowledge and preventive practice scores (S3 Table; Fig 2).

#### Knowledge.

Overall knowledge of dengue transmission was high. As the high knowledge score in Fig 2A shows, approximately 90% of respondents correctly identified mosquitoes as the sole vector of dengue virus transmission, indicating strong awareness of the basic disease etiology. This was consistent across both departments and sexes, although respondents with higher educational attainment generally achieved higher knowledge scores and were less likely to provide uncertain responses.

When asked about mosquito breeding sites (Fig 2B), the majority of participants recognized uncovered water containers as the primary risk. Over 74% correctly cited uncovered containers as breeding sites, and 62% could name two or more breeding site types, demonstrating a reasonably nuanced understanding.

Knowledge of preventive behaviors was somewhat more limited. While 62% mentioned emptying water containers and 42% cited cleaning water basins as effective strategies, only about half of the respondents named more than one measure (Fig 2C). This suggests that although knowledge of core concepts is widespread, detailed understanding and recall of diverse preventive strategies is less common, especially among those with lower education.

#### Attitudes.

Perceived vulnerability to dengue was mixed. Approximately one-third of participants considered their personal risk to be high, while another third considered it low. Notably, a gender difference emerged: 37% of women reported high perceived risk compared to only 22% of men. This subjective sense of risk was strongly associated with prior household experience of dengue, with 23% of all respondents reporting at least one dengue case in their household in the past two years (S4 Table).

When participants were asked about where they believed dengue transmission occurred, homes and public spaces (e.g., markets, parks) were cited far more frequently than workplaces or schools (S1 Fig). This reflects both the domestic breeding habits of *Aedes aegypti* and community perceptions of risk concentration in the household environment.

**Fig 3:**
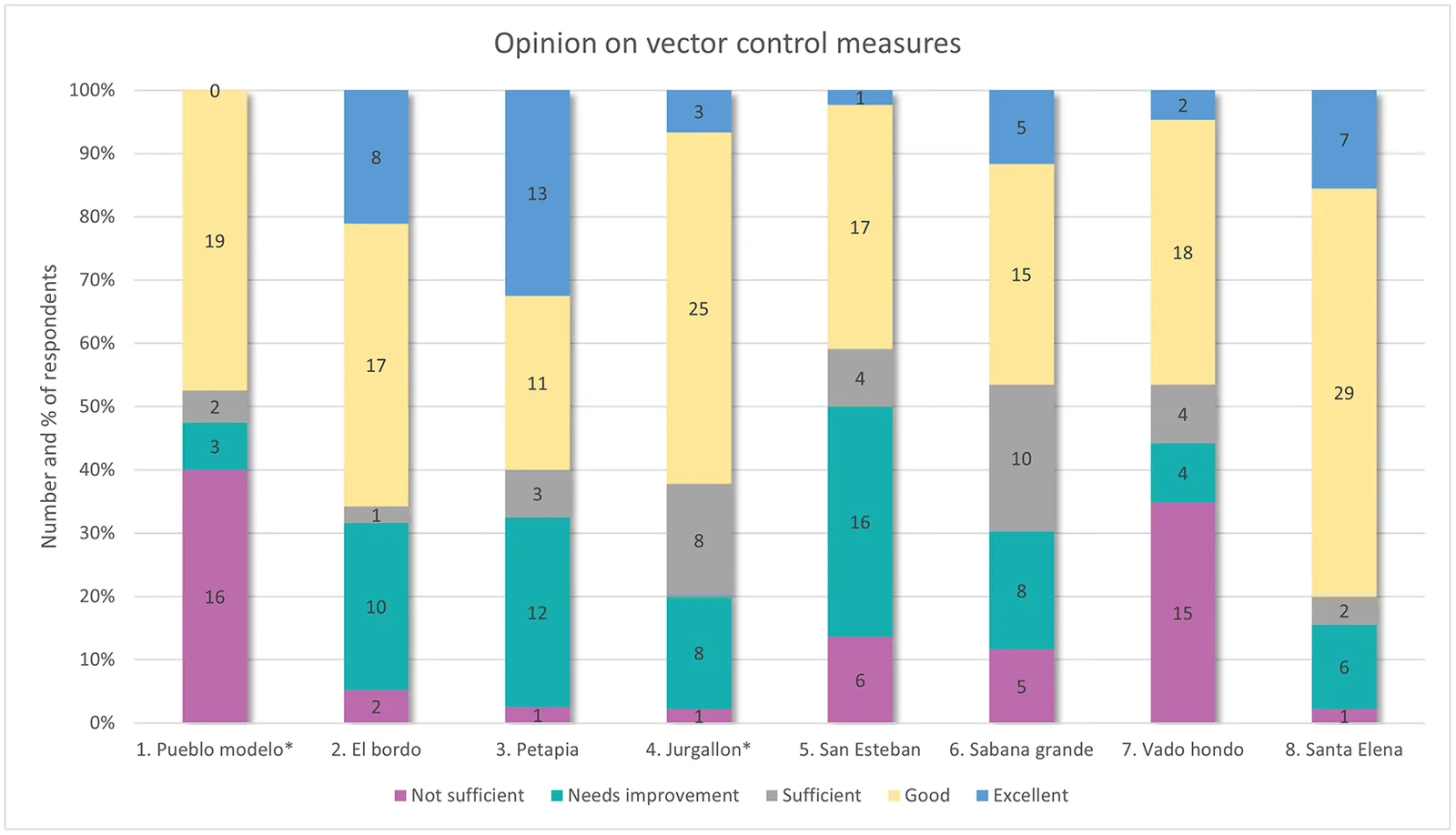
Opinion on vector control measures by locality. *Rural localities.

Satisfaction with local vector control efforts [larvicide: “Temephos”, space spraying, and source reduction] varied considerably by location (Fig 3). While 66.3% of respondents overall rated these efforts as sufficient, this figure ranged widely, from just 50% in San Esteban to 84% in Santa Elena (p < 0.001). Communities with lower satisfaction levels included Pueblo Modelo and Vado Hondo.

#### Attitudes toward copepod-based vector control.

**Fig 4:**
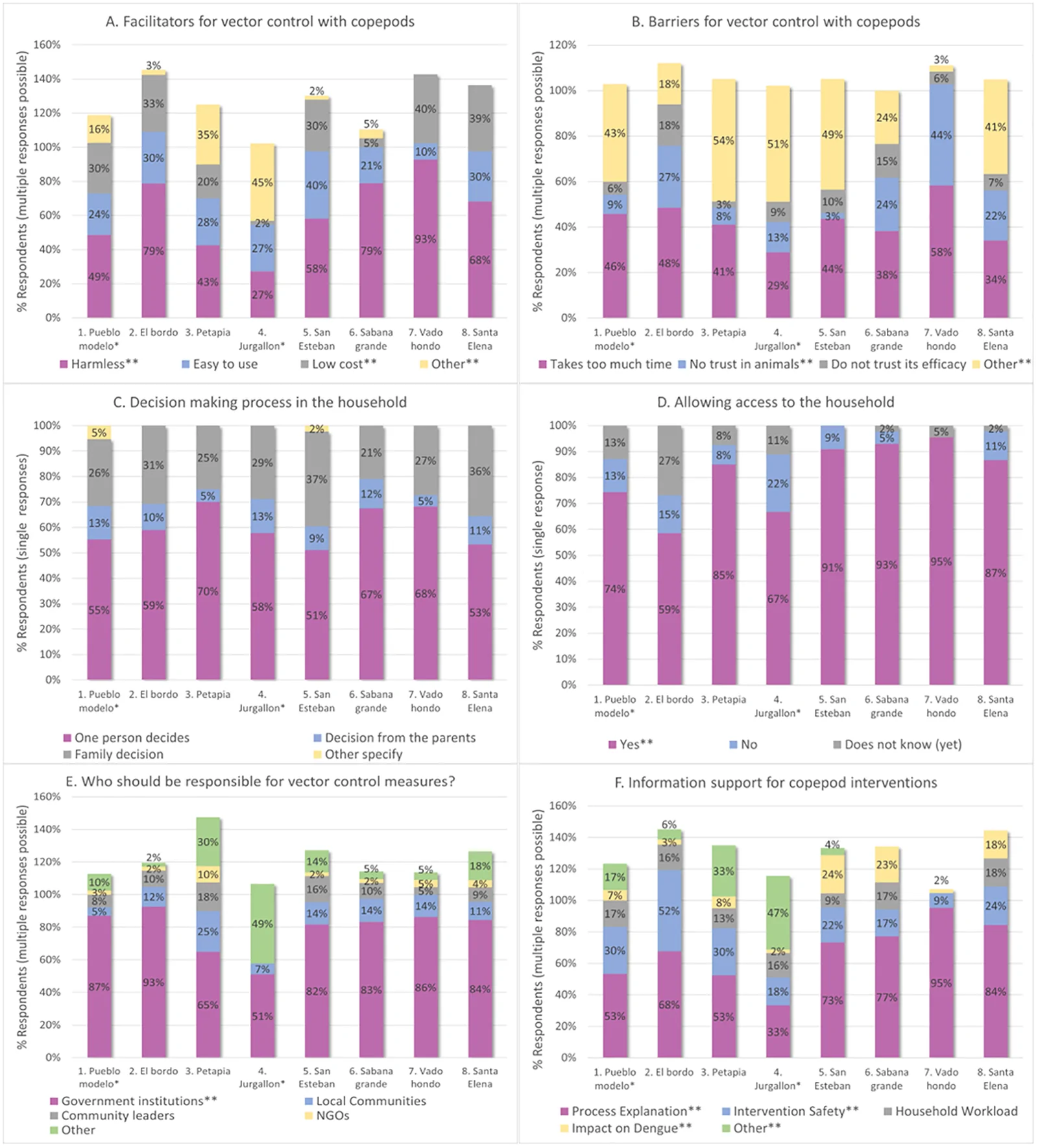
Community perceptions regarding the potential use of copepods for biological vector control by locality. Panels show: (A) perceived facilitators for copepod use, (B) perceived barriers and concerns, (C) household decision-making regarding mosquito control, (D) willingness to allow household access for copepod implementation, (E) perceived responsibility for vector control, and (F) information needs related to copepod use. *Rural localities. Statistical comparisons between communities were performed using chi-square tests.

To assess the community’s views on the feasibility of introducing copepods as a biological control method for *Aedes* mosquitoes, respondents answered a series of questions covering potential facilitators, perceived barriers, household decision-making, willingness to allow household access for implementation, perceptions of responsibility, and information needs. The results show marked variation across communities and are detailed in six sub-panels (Fig 4A–4F).

In response to the question on what would support their ability to use copepods, the majority of respondents across communities indicated that receiving information or training would be the most important facilitator. This response was particularly frequent in El Jurgallon and Pueblo Modelo, where over 80% of participants selected this option. Others mentioned the need for technical assistance or the presence of trained personnel (Fig 4A).

Concerns about the use of copepods were also reported. The most commonly cited barriers included uncertainty about water safety and a general lack of knowledge about the method. In more urbanized communities such as San Esteban and Sabana Grande, higher proportions of respondents expressed discomfort with introducing copepods into stored water. In contrast, rural communities, including El Bordo and El Jurgallon, reported lower levels of concern (Fig 4B).

When asked about household decision-making related to mosquito control, the majority of respondents indicated that such decisions were made jointly among family members. Some participants, particularly in rural areas, reported that decisions were made by a single individual, often the woman of the household (Fig 4C).

#### Practices.

Household practices related to mosquito control showed wide variation across respondents. As shown in Fig 2D, 37% of households reported taking no action to eliminate mosquito breeding sites. An additional 48% of respondents indicated they practiced only one preventive behavior. The most commonly reported measure was eliminating standing water, cited by 31% of respondents. Other actions, such as cleaning containers or covering water storage, were mentioned less frequently. Only a minority of respondents reported implementing multiple preventive strategies simultaneously. With regard to measures taken against adult mosquitoes, 25% of respondents indicated they took no preventive steps. Among those who did take action, the most frequently used method was insecticide spraying (28%), followed by use of mosquito bed nets (22%). The proportion of households employing more than three adult mosquito prevention measures was very low (Fig 2E).

Water was stored in over three-quarters of households and in open water containers for 61% of cases (Fig 2F). Only 23% of households never had to store water, 16% used covered containers only and 26% used both covered and uncovered containers.

Mosquito bed net usage was explored in more detail (Question C3). A majority of households (69%) reported not using any bed nets. Among those that did use them, nets were typically employed at night (30%). Use of bed nets during the daytime was less common, particularly for children under 12 years old. Among households with young children, night-time use was more frequent than daytime use.

**Fig 5:**
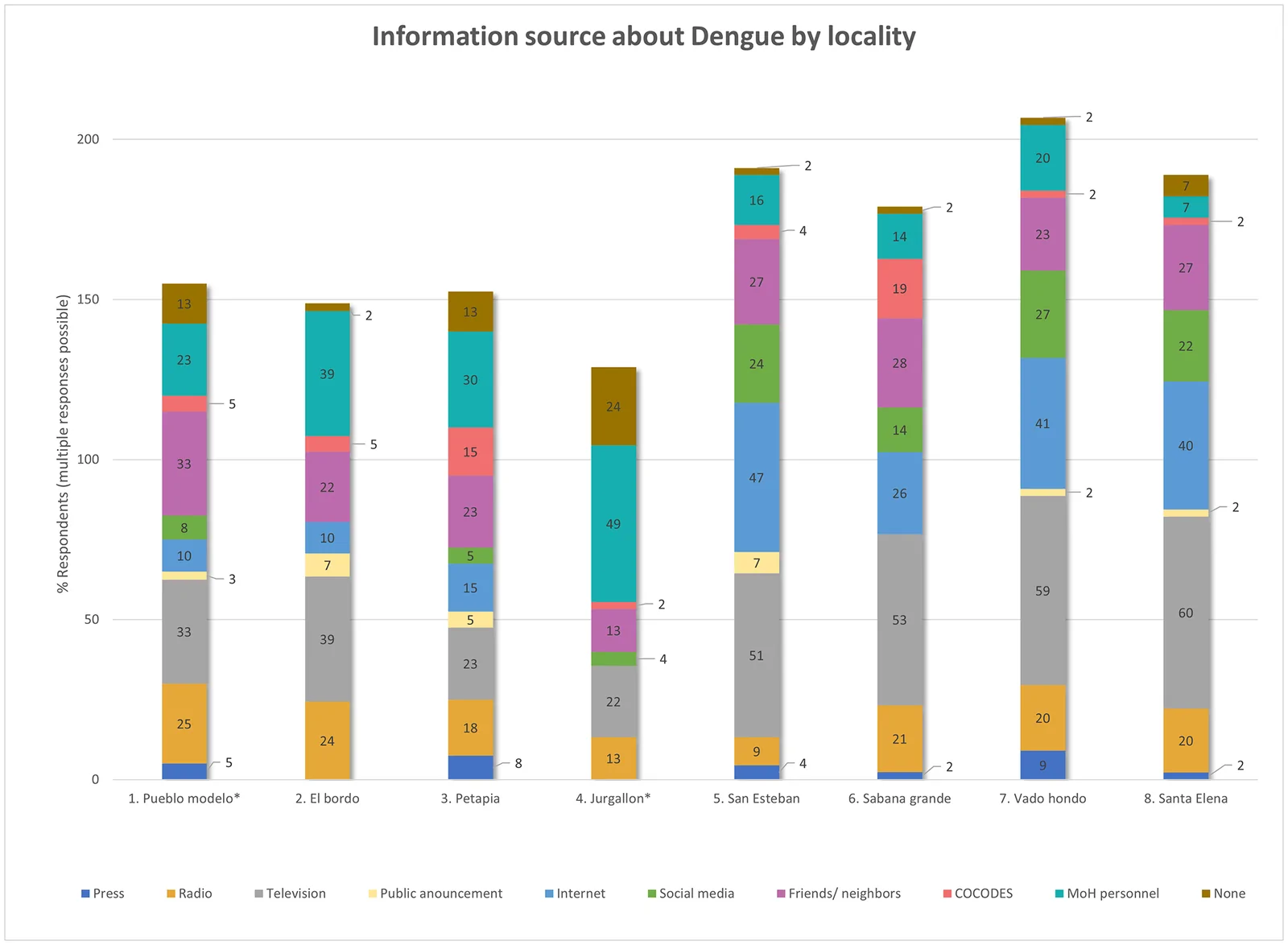
Information source about dengue by locality. *Rural localities.

Participants also reported their sources of information on dengue prevention. Television was the most widely cited medium, used by 86% of all respondents (Fig 5). Patterns of information-seeking varied by geography: communities in the southern part of Chiquimula more frequently used the Internet, while those in northern Chiquimula and Zacapa, especially in rural areas like El Jurgallon, relied more heavily on Ministry of Health staff and local health promoters for information.

### Regression

Exploratory regression analyses were conducted to assess associations between sociodemographic characteristics, household infrastructure variables, perceived dengue risk, and the different knowledge, attitude, and practice scores (Table 2). The variables sex, house ownership, outdoor household area, and energy source were initially evaluated but were not retained in the final exploratory models because they did not show statistically relevant associations during preliminary screening.

**Table 2:** Exploratory regression analyses of dengue-related knowledge (K), practice (P), and attitude (A) score outcomes in relation to selected sociodemographic and household variables. Coefficients from Poisson regression models are presented as log-rate estimates and coefficients from logistic regression models as log-odds estimates, each with 95% confidence intervals. Reference categories are indicated in the table with “(ref)”. Blank cells indicate variables not retained in the final model.

	K- Dengue Transmission Routes (B2)				K- Breeding sites (B3)				K- Preventive measures against mosquitoes (B4)			
Categories	Est.	95% CI		p^a^	Est.	95% CI		p^a^	Est.	95% CI		p^a^
**Education**												
Up to Primary school (ref)												
Secondary school and above	.103	.037	.169	.002 ***	.154	.045	.262	.006 ***	.193	.022	.364	.027 **
**Water access**												
Private piped water (ref)												
Municipal piped water	-.009	-.084	.066	.819	.220	.008	.432	.042 **	.333	.024	.642	.035 **
Other	-.077	-.145	-.008	.028 **	.367	.147	.586	.001 ***	.290	.179	.402	.000 ***
**Water storage**												
No (ref)												
Yes												
**Sanitation**												
Sewage (ref)												
Septic tank	.093	.037	.149	.001 ***	.046	-.165	.257	.667	.112	-.061	.286	.205
None or latrine	-.095	-.141	-.049	.000 ***	-.191	-.336	-.046	.010 ***	-.236	-.420	-.051	.012 **
**Perceived dengue risk**												
Low risk (ref)												
Some risk									.137	-.070	.344	.195
High risk									.192	.105	.278	.000 ***
**Constant**	1.674	1.611	1.738	.000 ***	.414	.365	.462	.000 ***	.167	.006	.327	.042 **
Up to Primary school (ref)												
Secondary school and above	.355	.176	.534	.000 ***	.275	.019	.531	.035 **				
**Water access**												
Private piped water (ref)												
Municipal piped water					.120	-.127	.367	.340				
Other					.288	.043	.532	.021 **				
**Water storage**												
No (ref)												
Yes	.371	-.082	.824	.108	.326	.108	.545	.003 ***				
**Sanitation**												
Sewage (ref)												
Septic tank					.109	-.162	.380	.430				
None or latrine					-.285	-.578	.009	.058 *				
**Perceived dengue risk**												
Low risk (ref)												
Some risk	-.258	-.466	-.049	.015 **					1.056	.317	1795	.005 ***
High risk	-.074	-.279	.131	.480					.547	-.056	1.15	.076 *
**Constant**	-.598	-1.082	-.115	.015 **	-.299	-.679	.080	.122	.977	.589	1366	.000 ***

^a^*** p-value <0.01, ** p-value <0.05, * p-value <0.1.

Higher educational attainment was consistently associated with higher knowledge and preventive practice scores. Household water access and sanitation indicators were also associated with several outcomes, particularly preventive practices related to mosquito breeding sites and adult mosquito control. Lower levels of sanitation infrastructure were associated with lower knowledge and prevention-related scores. In addition, moderate or high perceived dengue risk was associated with greater willingness to grant household access for potential vector control interventions.

## Discussion

This study investigated dengue-related knowledge, attitudes, and practices (KAP) in eight communities across eastern Guatemala and explored, for the first time in the region, perceptions of copepod-based biological control as an emerging vector management strategy. The findings revealed encouraging levels of basic knowledge about dengue transmission and breeding sites, but also highlighted significant inconsistencies in preventive practices and nuanced community attitudes toward innovative interventions. The study offers both a snapshot of current community engagement with dengue control and critical insights into the social and structural factors that may influence the feasibility of biological control in the Guatemalan context.

### Knowledge: Awareness exists, but depth varies

The high proportion of respondents who correctly identified mosquitoes as the exclusive vector of dengue aligns with findings from other Latin American countries where public awareness campaigns have been in place for decades. In Latin America and the Asia Pacific, studies have similarly shown widespread recognition of mosquito bites as the mode of transmission [13]. However, as in our study, this surface-level awareness often coexists with gaps in deeper or more actionable knowledge, such as understanding of mosquito breeding ecology or multi-step prevention strategies.

In our study, uncovered containers were widely recognized as breeding sites, and most respondents could name at least one preventive measure. Yet fewer were able to cite multiple interventions, and many defaulted to “I don’t know” when asked about less common strategies. This gradient of knowledge, particularly shaped by educational attainment, is well-documented in global KAP literature. Similar associations between educational attainment and dengue-related knowledge have been reported in community-based KAP studies from Latin America and Southeast Asia, where formal education was associated with improved recognition of mosquito breeding sites and preventive behaviors [11,14]. In Indonesia, for instance, Sulistyawati and colleagues [12] found that individuals with low literacy were significantly less likely to recognize correct larval habitats, even when they could describe symptoms of dengue. The observed educational divide in our study suggests that while basic messaging is reaching the population, more nuanced information, such as how daily behaviors contribute to vector proliferation, may not be effectively absorbed by those with limited formal schooling. This may reflect both the complexity of the information and the limitations of top-down communication strategies that do not account for local knowledge systems, preferred media channels, or visual literacy.

### Attitudes: Risk perception and trust in control measures

Perceptions of risk and responsibility are key drivers of behavior in any disease prevention effort. In our survey, only about one-third of participants perceived themselves at high risk of acquiring dengue, despite living in endemic areas. This echoes studies from Malaysia [15] and Colombia [14], which similarly report a disconnect between objective risk and perceived vulnerability. Personal experience with dengue, in the form of recent household cases, was the strongest correlate of high-risk perception in our sample, as in other Latin American settings. This tendency to underestimate risk in the absence of recent illness may contribute to lower engagement in preventive behaviors and highlights the challenge of sustaining community participation during inter-epidemic periods. It also underscores the challenge of sustaining long-term engagement with vector control in inter-epidemic periods. Notably, gender differences in risk perception were also evident in our study, with women more frequently reporting concern. This may reflect gendered roles in household management, caregiving and educational differences, and has been observed in other vector-borne disease contexts, such as Zika [16,17].

Satisfaction with government vector control efforts varied across communities, ranging from 50% to over 80%. These differences may reflect variability in service provision, but could also be influenced by community expectations, trust in institutions, and recent experiences with health authorities. In Puerto Rico, residents were found to prefer mosquito control programs applied at the neighborhood level and implemented by local governments [18]. The diversity of opinions regarding responsibility for vector control, ranging from full individual responsibility to complete reliance on public institutions, suggests a lack of shared ownership, which has been shown to hinder sustained community involvement. This fragmentation has been identified as a barrier in dengue programs and health service research [19,20].

### Practices: The Knowledge–action gap

Perhaps the most striking finding was the discrepancy between knowledge and action. Despite widespread awareness of breeding sites and transmission modes, 37% of households took no action to eliminate mosquito habitats, and only a small fraction adopted multiple simultaneous preventive measures. This gap between what people know and what they do is a common theme in vector control research and has been observed in various settings across the world [21,22]. However, behavioral practices should be interpreted cautiously, as self-reported KAP data may be influenced by recall bias and social desirability bias, particularly when respondents are asked about recommended preventive behaviors. Several explanations, such as attitude and motivation barriers [11,12], structural and resource limitations [23], and dependency on authorities [20] have been proposed in the literature. Structural barriers, such as irregular water supply and absence of waste management, can undermine people’s ability to implement what they know to be correct practices. In our study, such barriers were evident: frequent use of open water containers in areas lacking piped water, and limited coverage of covered storage systems, suggest that environmental constraints may limit behavioral options.

Time availability, perceived effectiveness of control measures, and the absence of visible mosquito presence during dry seasons may also contribute to limited engagement. Furthermore, the low use of mosquito nets, particularly during the day when *Aedes aegypti* is most active, indicates a misalignment between prevention strategies and entomological realities. Bed nets are often perceived as useful primarily against diseases transmitted by night-biting mosquitoes, such as malaria, but their potential use against diseases transmitted by day-biting Aedes mosquitoes, such as dengue, is often overlooked [24,25].

### Information sources and community communication channels

Television was the dominant source of dengue-related information across all communities, though southern localities reported higher use of internet sources, while northern rural areas relied more on Ministry of Health personnel. This spatial variation highlights the need for tailored communication strategies that account for media access and trust. In particular, reliance on passive media (TV, radio) may be insufficient to promote behavior change, especially for unfamiliar practices such as biological control. Previous programs have shown that there is no single best communication channel; rather, the effectiveness of different channels depends strongly on the sociocultural context and the recipients’ age, sex, and education [26,27]. In our study, the relatively low mention of local development councils (COCODES) as information sources suggests an underutilized opportunity for community-led messaging. Given the ubiquitous presence of COCODES across all study communities, these local governance structures may represent an underutilized platform for future community-based dengue prevention and biological vector control programs.

### Copepod-based control: A window of opportunity

A novel and important dimension of this study is the examination of community attitudes toward copepod-based biological control. While most respondents had no prior knowledge of copepods, the majority expressed openness to their use if clear information, technical guidance, and assurances about safety could be provided. This conditional acceptance is noteworthy given the unfamiliarity of the method in Guatemala and the tendency for biological control to raise questions about water quality and hygiene.

Interestingly, willingness to adopt copepods was higher in rural communities, where concerns about water safety were less frequently cited. This may reflect more frequent exposure to natural water sources, or greater familiarity with aquatic organisms in daily life. In contrast, urban residents were more likely to express skepticism, echoing findings from Vietnam, where urban acceptance of copepods was initially lower until extensive community engagement addressed concerns [28]. Participants were also clear in their information needs, ranging from practical maintenance to biological understanding, which suggests a high level of engagement and curiosity, rather than outright rejection. Importantly, the observed openness toward copepod-based control reflects perceived acceptability and willingness to consider the intervention under hypothetical future implementation conditions. These findings should not be interpreted as evidence of entomological effectiveness, operational feasibility, or long-term sustainability, which will require dedicated field-based implementation and evaluation studies. In this respect, Guatemalan communities may be well-positioned for pilot copepod interventions, especially if introduced through trusted community structures and supported by user-friendly, multilingual education materials.

What distinguishes the Vietnamese experience, and what Guatemala could potentially replicate, is the pairing of biological control with deep community ownership. In Vietnam, community volunteers were trained to monitor and replenish copepods, and local residents formed part of the implementation infrastructure. Such models could be adapted to the Guatemalan context, especially in rural areas where existing social networks (e.g., COCODES, appointed community leaders, women’s groups) can serve as implementation partners.

### Methodological strengths and limitations

This study has several strengths. It included communities from both urban and rural settings across two dengue-endemic departments in eastern Guatemala and combined conventional dengue KAP indicators with questions related to the acceptability of a novel biological vector control approach. The use of trained interviewers, pilot-testing of the questionnaire, and electronic data collection contributed to data consistency and completeness.

Several limitations should also be considered when interpreting the findings. First, the cross-sectional study design limits causal inference and reflects perceptions and practices only at a single point in time. Second, the household-based daytime recruitment strategy resulted in an overrepresentation of women, likely because women were more frequently present at home during interview hours. As gender may influence dengue risk perception and household prevention behaviors, this imbalance may have affected some estimates and limits generalizability to the broader adult population.

Third, the study was designed primarily for descriptive and exploratory purposes and did not aim to generate highly precise inferential estimates. No formal design effect adjustment was incorporated into the sample size calculation despite the cluster-based sampling approach. In addition, while the study included geographically diverse communities, the findings may not fully represent the socioeconomic, cultural, and infrastructural diversity of Guatemala as a whole.

Fourth, the study relied on self-reported information regarding preventive practices and perceptions, which may be affected by recall bias and social desirability bias. Participants may have overreported recommended preventive behaviors or underreported practices perceived negatively. Finally, although multivariable exploratory regression analyses were performed, residual and unmeasured confounding cannot be excluded.

## Conclusions and Implications for policy and practice

The findings of this study reflect communities that are aware of dengue and its risks, but not yet consistently translating this knowledge into preventive action. Structural barriers, uneven communication channels, and divergent perceptions of responsibility shape the behavioral landscape. Importantly, the study reveals that Guatemalan communities are open to exploring biological control via copepods, even in the absence of prior exposure, provided that trust, clarity, and local support are in place. In an era of increasing resistance to chemical larvicides and intensifying epidemic threats, the time is ripe to test and adapt biological strategies that have proven successful elsewhere. Copepod-based interventions, if co-created with communities and tailored to local realities, could become a valuable addition to Guatemala’s vector control toolkit. However, further field-based implementation studies are needed to evaluate their effectiveness, operational feasibility, long-term sustainability, and community uptake under real-world conditions.

## Supporting information

S1 Table Overview of housing characteristics by communities with absolute number and relative percentage computed per column and category. For each characteristic, the most common category is indicated in bold. * indicates a rural locality.
(DOCX)

S2 Table Study questions and response categories of all categorical questions with absolute number and relative percentage given. The total sample comprised N = 346 participants who provided consent. The score column indicates the type of response (y = yes, n = no, d = diverse based on actual “other response”) for which “1” was counted in the score. The maximum score for a given question is shown.
(DOCX)

S3 Table Scores as a function of sex and education (<2^nd^ = primary or no formal education, ≥ 2^nd^ = secondary education and above). (DOCX)

S4 Table Risk of dengue associated with reporting dengue in the household in the last 2 years. (DOCX)

S1 Fig Where is the risk of acquiring dengue (multiple responses possible). (DOCX)
