# Supplementary material for: Preparing for the tiny predators: Community readiness and perceptions of copepod-based dengue control in eastern guatemala
Source: PLoS Negl Trop Dis. 2026 Sep 3;20(9):e0014654. doi: 10.1371/journal.pntd.0014654 (PMC13541171; doi:10.1371/journal.pntd.0014654)
Supplement: Supplementary file 1 [file pntd.0014654.s001.docx]

**Supplementary table S1**

**Table S1.** Overview of housing characteristics by communities with absolute number and relative percentage computed per column and category. For each characteristic, in the most common category is indicated in bold. * indicate a rural locality.

| **Locality** (*=rural) (N)  **Characteristic** | **1 Pueblo Modelo* (N=42)** | | **2 El Bordo (N=42)** | | **3 Petapia (N=40)** | | **4 Jurgallon* (N=45)** | | **5 San Esteban (N=45)** | | **6 Sabana Grande (N=43)** | | **7 Vado Hondo (N=44)** | | **8 Santa Elena (N=45)** | |  | **Total (N=346)** | |
| --- | --- | --- | --- | --- | --- | --- | --- | --- | --- | --- | --- | --- | --- | --- | --- | --- | --- | --- | --- |
| **Household tenure** | N | % | N | % | N | % | N | % | N | % | N | % | N | % | N | % |  | N | % |
| **Owner** | 37 | **88.1** | 32 | **76.2** | 34 | **85.0** | 39 | **86.7** | 37 | **82,2** | 35 | **81.4** | 39 | **88.6** | 39 | **86.7** |  | 292 | **84.4** |
| Other | 5 | 11.9 | 10 | 23.8 | 6 | 15.0 | 6 | 13.3 | 8 | 17,78 | 8 | 18.6 | 5 | 11.4 | 6 | 13.3 |  | 54 | 15.6 |
| Total | 42 | 100 | 42 | 100 | 40 | 100 | 45 | 100 | 45 | 100 | 43 | 100 | 44 | 100 | 45 | 100 |  | 346 | 100 |
|  |  |  |  |  |  |  |  |  |  |  |  |  |  |  |  |  |  |  |  |
| **Household construction** | N | % | N | % | N | % | N | % | N | % | N | % | N | % | N | % |  | N | % |
| **Durable** | 20 | 47.6 | 38 | **90.5** | 38 | **95.0** | 25 | **55.6** | 41 | **91,11** | 36 | **83.7** | 43 | **97.7** | 44 | **97.8** |  | 285 | **82.4** |
| Other | 22 | **52.4** | 4 | 9.5 | 2 | 5.0 | 20 | 44.4 | 4 | 8,89 | 7 | 16.3 | 1 | 2.3 | 1 | 2.2 |  | 61 | 17.6 |
| Total | 42 | 100 | 42 | 100 | 40 | 100 | 45 | 100 | 45 | 100 | 43 | 100 | 44 | 100 | 45 | 100 |  | 346 | 100 |
|  |  |  |  |  |  |  |  |  |  |  |  |  |  |  |  |  |  |  |  |
| **Household construction** | N | % | N | % | N | % | N | % | N | % | N | % | N | % | N | % |  | N | % |
| **Concrete. brick. stone** | 20 | **47.6** | 38 | **90.5** | 38 | **95.0** | 25 | **55.6** | 41 | **91,11** | 36 | **83.7** | 43 | **97.7** | 44 | **97.8** |  | 285 | **82.4** |
| Adobe. wood | 9 | 21.4 | 2 | 4.8 | 0 | 0.0 | 13 | 28.9 | 4 | 8,9 | 7 | 16.3 | 1 | 2.3 | 0 | 0.0 |  | 36 | 10.4 |
| Metal sheet | 13 | 31.0 | 2 | 4.8 | 2 | 5.0 | 7 | 15.6 | 0 | 0 | 0 | 0 | 0 | 0.0 | 1 | 2.2 |  | 25 | 7.2 |
| Total | 42 | 100 | 42 | 100 | 40 | 100 | 45 | 100 | 45 | 100 | 43 | 100 | 44 | 100 | 45 | 100 |  | 346 | 100 |
|  |  |  |  |  |  |  |  |  |  |  |  |  |  |  |  |  |  |  |  |
| **Household outdoor area** | N | % | N | % | N | % | N | % | N | % | N | % | N | % | N | % |  | N | % |
| none | 1 | 2.4 | 0 | 0.0 | 1 | 2.5 | 2 | 4.4 | 6 | 13,3 | 2 | 4.65 | 1 | 2.3 | 5 | 11.1 |  | 18 | 5.2 |
| **Garden or Yard** | 41 | **97.6** | 42 | **100** | 38 | **95.0** | 42 | **93.3** | 38 | **84,4** | 41 | **95.35** | 43 | **97.7** | 36 | **80.0** |  | 321 | **92.8** |
| Terrace or balcony only | 0 | 0.0 | 0 | 0.0 | 1 | 2.5 | 1 | 2.2 | 1 | 2,2 | 0 | 0 | 0 | 0.0 | 4 | 8.9 |  | 7 | 2.0 |
| Total | 42 | 100 | 42 | 100 | 40 | 100 | 45 | 100 | 45 | 100 | 43 | 100 | 44 | 100 | 45 | 100 |  | 346 | 100 |
|  |  |  |  |  |  |  |  |  |  |  |  |  |  |  |  |  |  |  |  |
| **HH Type of Sanitation Service (toilet)** | N | % | N | % | N | % | N | % | N | % | N | % | N | % | N | % |  | N | % |
| Toilet connected to sewage network | 16 | 38.1 | 0 | 0.0 | 7 | 17.5 | 8 | 18.2 | 1 | 2,2 | 28 | **65.12** | 6 | 13.6 | 0 | 0.0 |  | 66 | 19.1 |
| **Toilet connected to septic tank** | 0 | 0.0 | 37 | **88.1** | 29 | **72.5** | 19 | **43.2** | 44 | **97,8** | 9 | 20.93 | 38 | **86.4** | 45 | **100** |  | 221 | **64.1** |
| Latrine or blind pit | 26 | **61.9** | 4 | 9.5 | 3 | 7.5 | 15 | 34.1 | 0 | 0,0 | 6 | 13.95 | 0 | 0.0 | 0 | 0.0 |  | 54 | 15.7 |
| No toilet | 0 | 0.0 | 1 | 2.4 | 1 | 2.5 | 2 | 4.6 | 0 | 0 | 0 | 0 | 0 | 0.0 | 0 | 0.0 |  | 4 | 1.2 |
| Total | 42 | 100 | 42 | 100 | 40 | 100 | 44 | 100 | 45 | 100 | 43 | 100 | 44 | 100 | 45 | 100 |  | 345 | 100 |
|  |  |  |  |  |  |  |  |  |  |  |  |  |  |  |  |  |  |  |  |
|  |  |  |  |  |  |  |  |  |  |  |  |  |  |  |  |  |  |  |  |
|  |  |  |  |  |  |  |  |  |  |  |  |  |  |  |  |  |  |  |  |
|  |  |  |  |  |  |  |  |  |  |  |  |  |  |  |  |  |  |  |  |
| **Household main water source** | N | % | N | % | N | % | N | % | N | % | N | % | N | % | N | % |  | N | % |
| **Piped water (private)** | 40 | **95.2** | 0 | 0.0 | 0 | 0.0 | 0 | 0.0 | 28 | **62,2** | 28 | **65.12** | 15 | **34.1** | 9 | 20.0 |  | 120 | **34.7** |
| **Piped water (municipal)** | 0 | 0.0 | 41 | **97.6** | 0 | 0.0 | 19 | **42.2** | 9 | 20,0 | 3 | 7.0 | 15 | **34.1** | 20 | **44.4** |  | 107 | **30.9** |
| Well (communal) | 1 | 2.4 | 1 | 2.4 | 36 | **90.0** | 3 | 6.7 | 7 | 15,6 | 11 | 25.58 | 0 | 0.0 | 1 | 2.2 |  | 60 | 17.3 |
| Well (private) | 1 | 2.4 | 0 | 0.0 | 3 | 7.5 | 13 | 28.9 | 0 | 0,0 | 0 | 0 | 0 | 0.0 | 12 | 26.7 |  | 29 | 8.4 |
| Water trucks | 0 | 0.0 | 0 | 0.0 | 0 | 0.0 | 9 | 20.0 | 0 | 0,0 | 0 | 0 | 0 | 0.0 | 1 | 2.2 |  | 10 | 2.9 |
| Piped water in public space | 0 | 0.0 | 0 | 0.0 | 0 | 0.0 | 0 | 0.0 | 0 | 0,0 | 0 | 0 | 14 | 31.8 | 0 | 0.0 |  | 14 | 4.1 |
| Other | 0 | 0.0 | 0 | 0.0 | 1 | 2.5 | 1 | 2.2 | 1 | 2,2 | 1 | 2.3 | 0 | 0.0 | 2 | 4.4 |  | 6 | 1.7 |
| Total | 42 | 100 | 42 | 100 | 40 | 100 | 45 | 100 | 45 | 100 | 43 | 100 | 44 | 100 | 45 | 100 |  | 346 | 100 |
|  |  |  |  |  |  |  |  |  |  |  |  |  |  |  |  |  |  |  |  |
| **Household energy source** | N | % | N | % | N | % | N | % | N | % | N | % | N | % | N | % |  | N | % |
| **Electrical power network** | 35 | **83.3** | 42 | 100 | 38 | 95.0 | 42 | 93.3 | 44 | 97,78 | 43 | **100** | 43 | 97.7 | 45 | **100** |  | 332 | **96.0** |
| Other | 7 | 16.7 | 0 | 0.0 | 2 | 5.0 | 3 | 6.7 | 1 | 2,22 | 0 | 0 | 1 | 2.3 | 0 | 0.0 |  | 14 | 4.1 |
| Total | 42 | 100 | 42 | 100 | 40 | 100 | 45 | 100 | 45 | 100 | 43 | 100 | 44 | 100 | 45 | 100 |  | 346 | 100 |
|  |  |  |  |  |  |  |  |  |  |  |  |  |  |  |  |  |  |  |  |
| **Are containers used to store water?** | N | % | N | % | N | % | N | % | N | % | N | % | N | % | N | % |  | N | % |
| No | 7 | 16.7 | 6 | 14.3 | 2 | 5.0 | 1 | 2.2 | 14 | 31,1 | 15 | 34.88 | 17 | 38.6 | 19 | 42.2 |  | 81 | 23.4 |
| **Yes** | 35 | **83.3** | 36 | **85.7** | 38 | **95.0** | 44 | **97.8** | 31 | **68.9** | 28 | **65.1** | 27 | **61.4** | 26 | **57.8** |  | 265 | **76.6** |
| Total | 42 | 100 | 42 | 100 | 40 | 100 | 45 | 100 | 45 | 100 | 43 | 100 | 44 | 100 | 45 | 100 |  | 346 | 100 |
| **Are containers used to store water?** | N | % | N | % | N | % | N | % | N | % | N | % | N | % | N | % |  | N | % |
| No | 7 | 16.7 | 6 | 14.3 | 2 | 5.0 | 1 | 2.2 | 14 | 31,1 | 15 | **34.88** | 17 | 38.6 | 19 | **42.2** |  | 81 | 23.4 |
| Containers with covers only | 9 | 21.4 | 7 | 16.7 | 10 | 25.0 | 3 | 6.7 | 9 | 20,0 | 4 | 9.3 | 5 | 11.4 | 8 | 17.8 |  | 55 | 15.9 |
| **Containers without covers only** | 24 | **57.1** | 18 | **42.9** | 15 | **37.5** | 29 | **64.4** | 18 | **40,0** | 11 | 25.6 | 1 | 2.3 | 2 | 4.4 |  | 118 | **34.1** |
| Both | 2 | 4.8 | 11 | 26.2 | 13 | 32.5 | 12 | 26.7 | 4 | 8,9 | 13 | 30.23 | 21 | **47.7** | 16 | 35.6 |  | 92 | 26.6 |
| Total | 42 | 100 | 42 | 100 | 40 | 100 | 45 | 100 | 45 | 100 | 43 | 100 | 44 | 100 | 45 | 100 |  | 346 | 100 |
|  |  |  |  |  |  |  |  |  |  |  |  |  |  |  |  |  |  |  |  |
