# Supplementary material for: Preparing for the tiny predators: Community readiness and perceptions of copepod-based dengue control in eastern guatemala
Source: PLoS Negl Trop Dis. 2026 Sep 3;20(9):e0014654. doi: 10.1371/journal.pntd.0014654 (PMC13541171; doi:10.1371/journal.pntd.0014654)
Supplement: Supplementary file 2 [file pntd.0014654.s002.docx]

**Table S2****.** Study questions and response categories of all categorical questions with absolute number and relative percentage given. The total sample consisted of N=346 entries where consent was given. The score column indicates the type of response (y= yes, n = no, d= diverse based on actual “other response”) for which “1” was counted in the score. The maximum score for a given question is shown.

| **Code** | **Questions and response categories (N=346)** | **N - Total** | **% - Total** | **Score** |
| --- | --- | --- | --- | --- |
| A6 | **What is the type of tenure of your HH? (single response)** |  |  |  |
| **A6_1** | Own | 292 | 84.4% |  |
| **A6_2** | Rented | 34 | 9.8% |  |
| **A6_3** | Rent free | 14 | 4.0% |  |
| **A6_4** | Communal property | 2 | 0.6% |  |
| **A6_5** | Does not know | 3 | 0.9% |  |
| **A6_6** | Other | 1 | 0.3% |  |
| A7 | **What material is your house made of? (structure, walls, and roof) (single response)** |  |  |  |
| **A7_1** | Strong structure: concrete, brick, block, stone, cement, asbestos, tile | 285 | 82.4% |  |
| **A7_2** | Adobe, bajareque, wood, straw, palm, earth, sticks, lepa, cane | 36 | 10.4% |  |
| **A7_3** | Metal Sheet | 25 | 7.2% |  |
| **A7_4** | Other |  |  |  |
| A8 | **What outdoor areas does your house have? (multiple response)** |  |  |  |
| A8_1 | None | 18 | 5.2% |  |
| A8_2 | Garden | 146 | 42.2% |  |
| A8_3 | Yard | 315 | 91.0% |  |
| A8_4 | Terrace | 22 | 6.4% |  |
| A8_5 | Balcony | 9 | 2.6% |  |
| A8_99 | Other |  |  |  |
| A9 | **What kind of sanitation facility does your HH have? (toilet) (single response)** |  |  |  |
| **A9_1** | Toilet connected to sewage network | 221 | 63.9% |  |
| **A9_2** | Toilet connected to septic tank | 66 | 19.1% |  |
| **A9_3** | Latrine or blind pit | 54 | 15.6% |  |
| **A9_4** | Does not have | 4 | 1.2% |  |
| **A9_5** | Other | 0 | 0.0% |  |
| A10 | **What is the main source of energy in your HH? (single response)** |  |  |  |
| **A10_1** | Electrical power network | 332 | 96.0% |  |
| **A10_2** | Solar or wind panel | 2 | 0.6% |  |
| **A10_3** | Natural gas | 1 | 0.3% |  |
| **A10_4** | Candle | 4 | 1.2% |  |
| **A10_5** | Does not know | 1 | 0.3% |  |
| **A10_6** | Other | 4 | 1.2% |  |
| A11 | **Which of the following communication and information services do you have access to? (multiple responses)** |  |  |  |
| A11_1 | Television | 299 | 86.4% |  |
| A11_2 | Radio | 214 | 61.8% |  |
| A11_3 | Cable | 175 | 50.6% |  |
| A11_4 | Telephone landline | 41 | 11.8% |  |
| A11_5 | Cellphone | 304 | 87.9% |  |
| A11_6 | Computer | 74 | 21.4% |  |
| A11_99 | Other | 4 | 1.2% |  |
| A12 | **How does your household obtain water for domestic use? (multiple responses)** |  |  |  |
| A12_1 | Municipal water pipes in the HH | 107 | 30.9% |  |
| A12_2 | Private water pipes in the HH | 120 | 34.7% |  |
| A12_3 | Municipal water pipes outside the HH | 1 | 0.3% |  |
| A12_4 | Water pipes outside the HH (Communal well or private service) | 14 | 4.0% |  |
| A12_5 | Public faucet | 5 | 1.4% |  |
| A12_6 | Own well | 35 | 10.1% |  |
| A12_7 | Drilled well | 4 | 1.2% |  |
| A12_8 | Communal well | 61 | 17.6% |  |
| A12_9 | Natural source (rain water, river, lake, spring) | 1 | 0.3% |  |
| A12_10 | Company that distributes water (trucks with water) | 16 | 4.6% |  |
| A12_99 | Other | 3 | 0.9% |  |
| A13 | **Do you ever store water inside or around the house? (multiple responses)** |  |  | max: 1 |
| A13_1 | Never | 82 | 23.7% | y |
| A13_2 | Containers with covers | 147 | 42.5% | y |
| A13_3 | Containers without covers | 202 | 58.4% |  |
| A13_99 | Other | 11 | 3.2% |  |
| B2 | **What do you think are the possible routes of dengue transmission? (multiple responses)** |  |  | max: 6 |
| B2_1 | Mosquito bite | 306 | 88.4% | y |
| B2_2 | Near a sick person | 5 | 1.4% | n |
| B2_3 | Through food | 1 | 0.3% | n |
| B2_4 | Trough water | 27 | 7.8% | n |
| B2_5 | By air | 0 | 0% | n |
| B2_6 | Does not know | 26 | 7.5% | 0 |
| B2_7 | Does not want to answer | 1 | 0.3% |  |
| B2_99 | Other | 5 | 1.4% | n |
|  |  |  |  |  |
| B3 | **Do you know what the typical breeding sites of dengue-transmitting mosquitoes are? (multiple responses)** |  |  | max: 7 |
| B3_1 | Uncovered containers | 258 | 74.6% | y |
| B3_2 | Solid waste (bottles, cans, plastic containers) | 55 | 15.9% | y |
| B3_3 | Water basins (e.g. *pilas*) or washing areas | 113 | 32.7% | y |
| B3_4 | Used tires | 57 | 16.5% | y |
| B3_5 | Puddles / stagnant water | 155 | 44.8% | y |
| B3_6 | Flowerpots and plants | 28 | 8.1% | y |
| B3_99 | Other | 7 | 2.0% |  |
| B3_7 | Does not know | 29 | 8.4% | 0 |
|  |  |  |  |  |
| B4 | **What can you do to avoid dengue mosquitoes breeding? (multiple responses)** |  |  | max: 7 |
| B4_1 | Empty water containers | 213 | 61.6% | y |
| B4_2 | Eliminate waste | 93 | 26.9% | y |
| B4_3 | Clean vegetation and shrubbery | 39 | 11.3% | y |
| B4_4 | Clean water basins or water tanks with brush | 146 | 42.2% | y |
| B4_5 | Use insecticide | 71 | 20.5% | y |
| B4_6 | Use window or door curtains | 22 | 6.4% | y |
| B4_7 | Does not know | 22 | 6.4% | 0 |
| B4_99 | Other | 20 | 5.8% | d |
|  |  |  |  |  |
| **B5** | **How do you obtain information about dengue and its prevention? (multiple responses)** |  |  |  |
| B5_1 | Newspaper | 13 | 3.8% |  |
| B5_2 | Radio | 64 | 18.5% |  |
| B5_3 | TV | 147 | 42.5% |  |
| B5_4 | Public advertisement | 11 | 3.2% |  |
| B5_5 | Internet | 82 | 23.7% |  |
| B5_6 | Social Media | 46 | 13.3% |  |
| B5_7 | neighbors and friends | 83 | 24.0% |  |
| B5_8 | COCODES (*Consejo comunitario de desarrollo*/ Community council for development) | 23 | 6.6% |  |
| B5_9 | Ministry of Health (MoH) personnel | 84 | 24.3% |  |
| B5_10 | Does not obtain information | 9 | 2.6% |  |
| B5_99 | Other | 28 | 8.1% |  |
|  |  |  |  |  |
| C1 | **Are you taking any measures to prevent mosquitoes from breeding? (multiple responses)** |  |  | max: 4 |
| C1_1 | No | 126 | 36.4% | 0 |
| C1_2 | Covering containers | 67 | 19.4% | y |
| C1_3 | Eliminating breeding sites | 107 | 30.9% | y |
| C1_4 | applying insecticide to containers | 45 | 13.0% | y |
| C1_99 | Other | 51 | 14.7% | d |
|  |  |  |  |  |
| C2 | **Are you taking any measures to control adult mosquitoes? (multiple responses)** |  |  | max: 11 |
| C2_1 | No | 85 | 24.6% | 0 |
| C2_2 | Mosquito nets in doors and windows | 36 | 10.4% | y |
| C2_3 | Protecting from mosquito biting during the day | 25 | 7.2% | y |
| C2_4 | Using repellents for mosquitos in the body | 35 | 10.1% | y |
| C2_5 | Bed nets | 75 | 21.7% | y |
| C2_6 | Spiral insecticide (e.g. “Autan”) | 54 | 15.6% | y |
| C2_7 | Laminated insecticide | 5 | 1.4% | y |
| C2_8 | Spray insecticide | 96 | 27.7% | y |
| C2_9 | Electric vapors | 17 | 4.9% | y |
| C2_10 | Fans /ventilators | 44 | 12.7% | y |
| C2_11 | Smoke | 21 | 6.1% | y |
| C2_99 | Other | 23 | 6.6% | d |
|  |  |  |  |  |
|  | **If you use mosquito nets, who uses them and when? (multiple responses)** |  |  |  |
| C3_1 | Under 12: Mosquito net use during the day | 26 | 7.5% |  |
| C3_2 | Under 12: Mosquito net use during the night | 76 | 22.0% |  |
| C3_3 | 12 and above: Mosquito net use during the day | 40 | 11.6% |  |
| C3_4 | 12 and above: Mosquito net use during the night | 103 | 29.7% |  |
|  | Total number of households using bed nets | 106 | 30.6% |  |
|  |  |  |  |  |
| D1 | **How much risk do you think you and your family are at of contracting dengue and becoming ill? (single response)** | | |  |
| **D1_1** | No risk | 36 | 10.4% |  |
| **D1_2** | Lower risk | 91 | 26.3% |  |
| **D1_3** | Some risk | 95 | 27.5% |  |
| **D1_4** | High risk | 113 | 32.7% |  |
| **D1_5** | Extremely high risk | 10 | 2.9% |  |
|  |  |  |  |  |
| D2 | **Have you or any member of your household been sick with dengue in the past two years? (single response)** | | | |
| **D2_1** | Yes | 79 | 22.8% |  |
| **D2_2** | No | 255 | 73.7% |  |
| **D2_3** | does not remember | 11 | 3.2% |  |
|  |  |  |  |  |
| D3 | **Where do you think you and your family are at the greatest risk of contracting dengue? (multiple responses)** |  |  |  |
| D3_1 | House | 188 | 54.3% |  |
| D3_2 | School | 75 | 21.7% |  |
| D3_3 | Work place | 77 | 22.3% |  |
| D3_4 | Public areas | 184 | 53.2% |  |
| D3_99 | Other | 29 | 8.4% |  |
|  |  |  |  |  |
| E1 | **What is your opinion of the vector-control methods currently offered by local health authorities? (single response)** |  |  |  |
| **E1_1** | Excellent | 39 | 11.3% |  |
| **E1_2** | Good | 151 | 43.6% |  |
| **E1_3** | Sufficient | 34 | 9.8% |  |
| **E1_4** | Needs to improve | 67 | 19.4% |  |
| **E1_5** | Not sufficient at all | 47 | 13.6% |  |
|  |  |  |  |  |
| E2 | **What would be important to you to accept and follow this new mosquito-larval control measure (copepods) (multiple responses)** |  |  |  |
| E2_1 | Harmless | 197 | 56.9% |  |
| E2_2 | Easy to use | 84 | 24.3% |  |
| E2_3 | Low cost | 80 | 23.1% |  |
| E2_99 | Other | 44 | 12.7% |  |
|  |  |  |  |  |
| E3 | **What factors would prevent you from implementing this new method (copepods) in your HH? (multiple responses)** |  |  |  |
| E3_1 | Takes too much time | 126 | 36.4% |  |
| E3_2 | Do not trust the animals | 55 | 15.9% |  |
| E3_3 | Do not trust its efficacy | 27 | 7.8% |  |
| E3_99 | Other | 110 | 31.8% |  |
|  |  |  |  |  |
| E4 | **What would be the decision-making process in your HH for accepting or rejecting this alternative biological control method (copepods)? (single response)** |  |  |  |
| **E4_1** | One person decides | 203 | 58.7% |  |
| **E4_2** | Decision from the parents | 33 | 9.5% |  |
| **E4_3** | Family decision | 98 | 28.3% |  |
| **E4_99** | Other specify | 3 | 0.9% |  |
|  |  |  |  |  |
| E5 | **Would you occasionally (twice a year) grant members of the mosquito-control team access to your home to gather information, inspect water-storage containers for mosquito larvae, and add copepods to those containers for larval control? (single response)** |  |  |  |
|  | Yes | 278 | 80.3% |  |
|  | No | 35 | 10.1% |  |
|  | Does not know (yet) | 28 | 8.1% |  |
| E6 | **Who do you think should be responsible for the routine implementation of this mosquito-control measures (copepods)? (multiple responses)** |  |  |  |
| E6_1 | Government institutions | 268 | 77.5% |  |
| E6_2 | Local Communities | 43 | 12.4% |  |
| E6_3 | Community leaders | 31 | 9.0% |  |
| E6_4 | NGOs | 12 | 3.5% |  |
| E6_99 | Other | 57 | 16.5% |  |
|  |  |  |  |  |
| E7 | **What information would you like to have to support this type of intervention (copepods) in your HH? (multiple responses)** |  |  |  |
| E7_1 | How it works | 212 | 61.3% |  |
| E7_2 | Safety of intervention | 76 | 22.0% |  |
| E7_3 | Workload for the household | 40 | 11.6% |  |
| E7_4 | Impact on dengue | 35 | 10.1% |  |
| E7_99 | Other | 43 | 12.4% |  |
|  |  |  |  |  |
