# Supplementary material for: Preparing for the tiny predators: Community readiness and perceptions of copepod-based dengue control in eastern guatemala
Source: PLoS Negl Trop Dis. 2026 Sep 3;20(9):e0014654. doi: 10.1371/journal.pntd.0014654 (PMC13541171; doi:10.1371/journal.pntd.0014654)
Supplement: Supplementary file 3 [file pntd.0014654.s003.docx]

**Table S3**. Scores as a function of sex and education (<2^nd^ = primary or no formal education, **≥**2^nd^ = secondary education and above)

| **SCORE** | **Scores (max)** | **Female** | **Male** | **Total** | **p** |  | **Educ <**2^nd^ | **Educ ≥**2^nd^ | **Total** | **p** |
| --- | --- | --- | --- | --- | --- | --- | --- | --- | --- | --- |
|  | **KNOWLEDGE** |  |  |  |  |  |  |  |  |  |
| B2 | **Dengue Transmission Routes (max=6)** | **Female** | **Male** | **Total** | **p** |  | **Educ <**2^nd^ | **Educ ≥**2^nd^ | **Total** | **p** |
| 0 | N | 19 | 6 | 25 | 0.8613 |  | 24 | 2 | 26 | **0.0130** |
|  | % | 7.1 | 7.9 | 7.3 |  |  | 11.2 | 1.6 | 7.6 |  |
| 4 | N | 8 | 1 | 9 |  |  | 5 | 4 | 9 |  |
|  | % | 3 | 1.3 | 2.6 |  |  | 2.3 | 3.1 | 2.6 |  |
| 5 | N | 22 | 7 | 29 |  |  | 17 | 12 | 29 |  |
|  | % | 8.3 | 9.2 | 8.5 |  |  | 7.9 | 9.3 | 8.4 |  |
| 6 | N | 217 | 62 | 279 |  |  | 169 | 111 | 280 |  |
|  | % | 81.6 | 81.6 | 81.6 |  |  | **78.6** | **86.1** | **81.4** |  |
|  | Total _N | 266 | 76 | 342 |  |  | 215 | 129 | 344 |  |
|  | Total % | 100 | 100 | 100 |  |  | 100 | 100 | 100 |  |
| B3 | **Breeding sites (max=7)** | **Female** | **Male** | **Total** | **p** |  | **Educ <**2^nd^ | **Educ ≥**2^nd^ | **Total** | **p** |
| 0 | N | 22 | 7 | 29 | 0.6422 |  | 25 | 5 | 30 | **0.0205** |
|  | % | 8.4 | 9.2 | 8.6 |  |  | 11.6 | 4 | 8.8 |  |
| 1 | N | 71 | 27 | 98 |  |  | 65 | 34 | 99 |  |
|  | % | 27 | 35.5 | 28.9 |  |  | 30.2 | 27 | 29 |  |
| 2 | N | 98 | 23 | 121 |  |  | 74 | 47 | 121 |  |
|  | % | 37.3 | 30.3 | 35.7 |  |  | **34.4** | **37.3** | **35.5** |  |
| 3 | N | 49 | 15 | 64 |  |  | 37 | 26 | 63 |  |
|  | % | 18.6 | 19.7 | 18.9 |  |  | 17.2 | 20.6 | 18.5 |  |
| 4 | N | 13 | 3 | 16 |  |  | 7 | 10 | 17 |  |
|  | % | 4.9 | 4 | 4.7 |  |  | 3.3 | 7.9 | 5 |  |
| 5 | N | 4 | 1 | 5 |  |  | 5 | 0 | 5 |  |
|  | % | 1.5 | 1.3 | 1.5 |  |  | 2.3 | 0 | 1.5 |  |
| 6 | N | 6 | 0 | 6 |  |  | 2 | 4 | 6 |  |
|  | % | 2.3 | 0 | 1.8 |  |  | 0.9 | 3.2 | 1.8 |  |
|  | Total _N | 263 | 76 | 339 |  |  | 215 | 126 | 341 |  |
|  | Total % | 100 | 100 | 100 |  |  | 100 | 100 | 100 |  |
| B4 | **Preventive measures (max=7)** | **Female** | **Male** | **Total** | **p** |  | **Educ <**2^nd^ | **Educ ≥**2^nd^ | **Total** | **p** |
| 0 | N | 21 | 5 | 26 | 0.9772 |  | 24 | 3 | 27 | **0.0052** |
|  | % | 7.9 | 6.8 | 7.7 |  |  | 11.2 | 2.4 | 7.9 |  |
| 1 | N | 102 | 32 | 134 |  |  | 82 | 52 | 134 |  |
|  | % | 38.5 | 43.2 | 39.5 |  |  | **38.1** | **41.3** | **39.3** |  |
| 2 | N | 88 | 24 | 112 |  |  | 76 | 36 | 112 |  |
|  | % | 33.2 | 32.4 | 33 |  |  | 35.4 | 28.6 | 32.8 |  |
| 3 | N | 37 | 9 | 46 |  |  | 24 | 22 | 46 |  |
|  | % | 14 | 12.2 | 13.6 |  |  | 11.2 | 17.5 | 13.5 |  |
| 4 | N | 12 | 3 | 15 |  |  | 8 | 8 | 16 |  |
|  | % | 4.5 | 4.1 | 4.4 |  |  | 3.7 | 6.4 | 4.7 |  |
| 5 | N | 3 | 1 | 4 |  |  | 1 | 3 | 4 |  |
|  | % | 1.1 | 1.4 | 1.2 |  |  | 0.5 | 2.4 | 1.2 |  |
| 6 | N | 2 | 0 | 2 |  |  | 0 | 2 | 2 |  |
|  | % | 0.8 | 0 | 0.6 |  |  | 0 | 1.6 | 0.6 |  |
|  | Total _N | 265 | 74 | 339 |  |  | 215 | 126 | 341 |  |
|  | Total % | 100 | 100 | 100 |  |  | 100 | 100 | 100 |  |
|  | **PRACTICES** |  |  |  |  |  |  |  |  |  |
| C1 | **Preventive measures against breeding sites (max=4)** | **Female** | **Male** | **Total** | **p** |  | **Educ <**2^nd^ | **Educ ≥**2^nd^ | **Total** | **p** |
| 0 | N | 98 | 26 | 124 | 0.9399 |  | 93 | 33 | 126 | **0.0036** |
|  | % | 37.6 | 35.1 | 37 |  |  | 44.1 | 26.2 | 37.4 |  |
| 1 | N | 126 | 36 | 162 |  |  | 95 | 67 | 162 |  |
|  | % | 48.3 | 48.7 | 48.4 |  |  | 45 | 53.2 | 48.1 |  |
| 2 | N | 35 | 11 | 46 |  |  | 21 | 25 | 46 |  |
|  | % | 13.4 | 14.9 | 13.7 |  |  | 10 | 19.8 | 13.7 |  |
| 3 | N | 2 | 1 | 3 |  |  | 2 | 1 | 3 |  |
|  | % | 0.8 | 1.4 | 0.9 |  |  | 1 | 0.8 | 0.9 |  |
|  | Total _N | 261 | 74 | 335 |  |  | 211 | 126 | 337 |  |
|  | Total % | 100 | 100 | 100 |  |  | 100 | 100 | 100 |  |
| C2 | **Preventive measures against adult mosquitoes (max=11)** | **Female** | **Male** | **Total** | **p** |  | **Educ <**2^nd^ | **Educ ≥**2^nd^ | **Total** | **p** |
| 0 | N | 64 | 23 | 87 | 0.7687 |  | 68 | 19 | 87 | **0.0230** |
|  | % | 24.2 | 30.3 | 25.5 |  |  | 31.8 | 14.7 | 25.4 |  |
| 1 | N | 113 | 31 | 144 |  |  | 83 | 61 | 144 |  |
|  | % | 42.6 | 40.8 | 42.2 |  |  | 38.8 | 47.3 | 42 |  |
| 2 | N | 58 | 17 | 75 |  |  | 44 | 32 | 76 |  |
|  | % | 21.9 | 22.4 | 22 |  |  | 20.6 | 24.8 | 22.2 |  |
| 3 | N | 24 | 4 | 28 |  |  | 16 | 13 | 29 |  |
|  | % | 9.1 | 5.3 | 8.2 |  |  | 7.5 | 10.1 | 8.5 |  |
| 4 | N | 2 | 1 | 3 |  |  | 2 | 1 | 3 |  |
|  | % | 0.8 | 1.3 | 0.9 |  |  | 0.9 | 0.8 | 0.9 |  |
| 5 | N | 3 | 0 | 3 |  |  | 1 | 2 | 3 |  |
|  | % | 1.1 | 0 | 0.9 |  |  | 0.5 | 1.6 | 0.9 |  |
| 6 | N | 1 | 0 | 1 |  |  | 0 | 1 | 1 |  |
|  | % | 0.4 | 0 | 0.3 |  |  | 0 | 0.8 | 0.3 |  |
|  | Total _N | 265 | 76 | 341 |  |  | 214 | 129 | 343 |  |
|  | Total % | 100 | 100 | 100 |  |  | 100 | 100 | 100 |  |
| A13 | **Water container used to store water (max=1)** | **Female** | **Male** | **Total** | **p** |  | **Educ <**2^nd^ | **Educ ≥**2^nd^ | **Total** | **p** |
| 0 (open) | N | 157 | 49 | 206 | 0.392 |  | 131 | 78 | 209 | 0.932 |
|  | % | 59.0 | 64.5 | 60.2 |  |  | 60.9 | 60.5 | 60.8 |  |
| 1 (no or covered) | N | 109 | 27 | 136 |  |  | 84 | 51 | 135 |  |
|  | % | 41.0 | 35.5 | 39.8 |  |  | 39.1 | 39.5 | 39.2 |  |
|  | Total _N | 266 | 76 | 342 |  |  | 215 | 129 | 344 |  |
|  | Total % | 100 | 100 | 100 |  |  | 100 | 100 | 100 |  |
|  |  |  |  |  |  |  |  |  |  |  |
