# Supplementary material for: Preparing for the tiny predators: Community readiness and perceptions of copepod-based dengue control in eastern guatemala
Source: PLoS Negl Trop Dis. 2026 Sep 3;20(9):e0014654. doi: 10.1371/journal.pntd.0014654 (PMC13541171; doi:10.1371/journal.pntd.0014654)
Supplement: Supplementary file 4 [file pntd.0014654.s004.docx]

**Table S4.** Risk of dengue associated with reporting dengue in the household in the last 2 years

| **Perceived dengue risk** | **No** | |  | **Yes** | |  | **Total** | | **P value** |
| --- | --- | --- | --- | --- | --- | --- | --- | --- | --- |
|  | N | row %  (column %) |  | N | row %  (column %) |  | N | row %  (column %) | **0.001** |
| Low risk | 113 | 88,3% |  | 15 | 11,7% |  | 128 | 100% |  |
|  |  | (42,3%) |  |  | (19,0%) |  |  | (37,0%) |  |
|  |  |  |  |  |  |  |  |  |  |
| Some risk | 69 | 72,6% |  | 26 | 27,4% |  | 95 | 100% |  |
|  |  | (25,8%) |  |  | (32,9%) |  |  | (27,5%) |  |
|  |  |  |  |  |  |  |  |  |  |
| High risk | 85 | 69,1% |  | 38 | 30,9% |  | 123 | 100% |  |
|  |  | (31,8%) |  |  | (48,1%) |  |  | (35,6%) |  |
|  |  |  |  |  |  |  |  |  |  |
| Total | 267 | 77,2% |  | 79 | 22,8% |  | 346 | 100% |  |
|  |  | (100%) |  |  | (100%) |  |  | (100%) |  |
