# Supplementary material for: Preparing for the tiny predators: Community readiness and perceptions of copepod-based dengue control in eastern guatemala
Source: PLoS Negl Trop Dis. 2026 Sep 3;20(9):e0014654. doi: 10.1371/journal.pntd.0014654 (PMC13541171; doi:10.1371/journal.pntd.0014654)
Supplement: Supplementary file 6 [file pntd.0014654.s007.docx]

**Full title: Preparing for the Tiny Predators: Community Readiness and Perceptions of Copepod-Based Dengue Control in Eastern Guatemala**

**Short title: Community Readiness for Copepod-Based Dengue Control in Eastern Guatemala**

**Authors**

Valérie R Louis, Carlos Alberto Montenegro-Quinoñez, Till Bärnighausen, Peter Dambach

**Response to Reviewers/Editors**

We greatly appreciate the constructive and insightful comments provided by the Editor and Reviewers. Their feedback has substantially improved the clarity, methodological transparency, and interpretability of our study.

We have carefully revised the manuscript in response to all comments. Major revisions include:

- clarification of the sampling strategy and sample size considerations,
- expanded explanation of questionnaire development, translation, and scoring procedures,
- additional detail on statistical analyses, including justification of regression approaches and handling of missing data,
- clarification of the interpretation of regression coefficients,
- improved presentation of tables and figures,
- expanded discussion of methodological limitations and potential biases,
- strengthened distinction between community acceptance and intervention effectiveness,
- clarification of ethical procedures and confidentiality measures,
- revision of conclusions and data presentation throughout the manuscript.

All changes have been incorporated into the revised manuscript and are highlighted in the tracked-changes version.

**Response to the Editor**

**Editor Comment:**

“I would like to ask authors to pay careful attention to the important concerns reported by Reviewers specially regarding the analysis and ethics/consent.”

**Response:**

We thank the Editor for this guidance. We carefully revised the manuscript with particular attention to methodological transparency, statistical analysis, and ethical considerations. Detailed clarifications have been added regarding sample size considerations, clustered sampling, questionnaire development and translation, score construction, regression modeling, missing data handling, and confidentiality procedures. We also substantially expanded the limitations section to address representativeness, potential biases, and residual confounding.

**Journal Requirement: CRediT author contributions**

**Response:**

The CRediT author contribution statements have been updated in the submission system to fully reflect the contributions of all authors.

**Journal Requirement: Figure 1 copyright and basemap source**

**Response:**

We thank the editorial office for highlighting this issue. Figure 1 has been revised to use an openly accessible basemap (Copernicus Sentinel 2 satellite imagery) compatible with the journal’s CC BY requirements. The source and licensing information of the shapefile/basemap have been added to the figure legend.

Basemap shapefile obtained from United Nations Office for the Coordination of Humanitarian Affairs (OCHA), (https://data.humdata.org/dataset/cod-ab-gtm). Background imagery derived from Copernicus Sentinel-2 satellite data accessed through the Sentinel Hub EO Browser (https://apps.sentinel-hub.com/eo-browser/). Copernicus Sentinel data are provided on a free and open basis under the Copernicus data policy.

**Journal Requirement: Data availability**

**Response:**

We acknowledge the journal’s data availability requirements. The data that we had indicated as being available upon request through the corresponding author is actually already available and was uploaded during the initial submission (File name: Supplementary Table_Figures). The Data Availability Statement has been revised accordingly.

**Reviewer #1**

We thank Reviewer #1 for the positive assessment of the study objectives and overall design, as well as for the detailed and constructive comments.

**Comment:**

“The methodology for calculating the compound score is confusing and needs more clarification for the reader.”

**Response:**

We agree and have substantially revised the description of score construction in the Methods section. We now explicitly explain how scores were generated, how correct responses were defined, and the maximum achievable score for each score category. We also clarified that the scoring system was based on predefined expert criteria.

**Changes made:**

- Expanded explanation of compound score generation in the Methods section.
- Added clearer description of score ranges and interpretation.
- Clarified score construction in Table S2 and referenced it more explicitly in the main text.

**Comment:**

“The use of stepwise regression … is generally discouraged…”

**Response:**

We appreciate this important point. We revised the manuscript to frame the regression analyses as exploratory rather than confirmatory. We also clarified that the stepwise procedure was used only as a preliminary variable selection tool due to the exploratory nature of the study and the relatively large number of potential explanatory variables compared to sample size.

**Changes made:**

- Added justification for exploratory variable selection.
- Revised wording throughout the manuscript to avoid overinterpretation of inferential findings.
- Expanded the limitations section to acknowledge limitations associated with stepwise selection procedures.

**Comment:**

“Please provide more details on missing data.”

**Response:**

We have added additional information on missing data handling. Missing responses were generally infrequent and were handled using complete-case analysis for the respective variables. We now explicitly report that denominators varied slightly between analyses due to item non-response.

**Changes made:**

- Expanded Methods section regarding missing data.
- Clarified complete-case approach and varying denominators.

**Comment:**

“Please confirm that the final models did not exhibit overdispersion…”

**Response:**

We thank the reviewer for this suggestion. We assessed model dispersion and confirmed that substantial overdispersion was not observed in the final Poisson models.

**Changes made:**

- Added statement in the Analysis section confirming that overdispersion diagnostics were assessed and did not indicate major violations of model assumptions.

**Comment:**

“Please provide more details on the purpose of using vce(cluster ‘community’).”

**Response:**

We expanded the statistical methods section to clarify that clustered standard errors were used to account for non-independence of observations within communities and potential intra-community correlation arising from the cluster-based sampling design.

**Changes made:**

- Added explanation of clustered standard errors in the Methods section.

**Comment:**

“Please indicate if ethical approval was obtained from the institution where data was analyzed.”

**Response:**

We clarified the ethical approval procedures and specified the approving institution more clearly.

**Changes made:**

- Expanded Ethics and Consent section accordingly.

**Comment:**

“The regression table should explicitly state reference categories.”

**Response:**

We agree and revised Table 2 to explicitly identify all reference categories.

**Changes made:**

- Reference categories are now clearly marked in Table 2.
- Table captions were expanded for clarity.

**Comment:**

“The estimates represent log-rate ratios/log-odds ratios and should be clearly stated.”

**Response:**

We thank the reviewer for this observation. The manuscript now clearly explains the interpretation of coefficients from Poisson and logistic regression models.

**Changes made:**

- Added clarification in the Analysis and Results sections regarding interpretation of regression estimates.

**Comment:**

“Community acceptance does not equate to effectiveness.”

**Response:**

We fully agree and strengthened the Conclusions section accordingly.

**Changes made:**

- Added explicit statement emphasizing that community acceptance represents social feasibility only and that entomological and implementation effectiveness still require rigorous field evaluation.

**Reviewer #2**

We thank Reviewer #2 for the detailed and thoughtful review and for recognizing the public health relevance and novelty of the study.

**Comment:**

“The sample size calculation … clustered sampling design was used without accounting for a design effect…”

**Response:**

We appreciate this important point. We revised the sample size section to acknowledge that no formal design effect was incorporated and clarified that the study was primarily designed to provide descriptive and exploratory insights rather than highly precise inferential estimates.

**Changes made:**

- Revised Sample Size subsection accordingly.
- Added clarification regarding exploratory interpretation and precision limitations.

**Comment:**

“Clarify how the team ensured even household coverage and random starting positions.”

**Response:**

We expanded the description of the household sampling strategy and clarified that random starting points were selected before systematic sampling was applied.

**Changes made:**

- Expanded Survey Population subsection.

**Comment:**

“Please cite validated KAP surveys.”

**Response:**

We added references to validated dengue KAP survey instruments that informed questionnaire development.

**Changes made:**

- Added references and citations in the Questionnaire and Data Collection section (The reference numbers themselves don´t appear in track change mode because this caused problems with Zotero when we tried it)

**Comment:**

“Please clarify translation and back-translation procedures.”

**Response:**

We expanded the manuscript to clarify the translation process and questionnaire review procedures.

**Changes made:**

- Added description of Spanish translation, review by bilingual researchers, and piloting procedures.

**Comment:**

“Please mention how descriptive statistics were presented.”

**Response:**

We added clarification that descriptive statistics were summarized using frequencies, percentages, means, and standard deviations as appropriate.

**Changes made:**

- Added detail in Analysis section.

**Comment:**

“Please specify that correctness was based on predefined criteria.”

**Response:**

We agree and revised the wording accordingly.

**Changes made:**

- Clarified expert-defined scoring criteria in Methods section.

**Comment:**

“What factors were model selection based on?”

**Response:**

The models were selected based on exploratory variable screening, epidemiological relevance, and statistical significance within the exploratory framework.

**Changes made:**

- Clarified model-building strategy in the Methods section.

**Comment:**

“What was the outcome estimate?”

**Response:**

We clarified the interpretation of regression coefficients and specified the type of model outputs produced.

**Changes made:**

- Added clarification in Methods and Results.

**Comment:**

“Explain how confidentiality was strictly maintained.”

**Response:**

We expanded the Ethics section to explain anonymization procedures, restricted access to data, and separation of identifiers from survey responses.

**Changes made:**

- Revised Ethics and Consent section.

**Comment:**

“Several results statements belong in the discussion.”

**Response:**

We agree and revised the Results section to focus on descriptive findings only. Interpretative statements were moved to the Discussion section.

**Changes made:**

- Revised Results section throughout.

**Comment:**

“Table 1 percentages and p-values require correction.”

**Response:**

We corrected Table 1 formatting and changed the percentage calculations so that not male and female sum up to 100% but all types within one category, e.g. education). We also removed unnecessary inferential statistics from descriptive tables.

**Changes made:**

- Revised Table 1 accordingly.

**Comment:**

“Please specify how perception data were collected.”

**Response:**

We added additional detail regarding perception-related survey items and response formats.

**Changes made:**

- Expanded Methods section accordingly.

**Comment:**

“Please include all statistical analyses conducted in supplementary files.”

**Response:**

Additional statistical outputs and supplementary analytical details have been added to the supplementary materials.

**Changes made:**

- Supplementary materials expanded.

**Comment:**

“Address recruitment limitations, recall bias, social desirability bias, and residual confounding.”

**Response:**

We substantially expanded the limitations section to address these important methodological considerations.

**Changes made:**

- Added discussion of:
  - overrepresentation of women,
  - daytime recruitment limitations,
  - representativeness constraints,
  - recall bias,
  - social desirability bias,
  - residual and unmeasured confounding.

**Comment:**

“Figure 4 caption is incomplete.”

**Response:**

We corrected and expanded the figure caption.

**Changes made:**

- Revised Figure 4 caption.
