# Supplementary material for: Preparing for the tiny predators: Community readiness and perceptions of copepod-based dengue control in eastern guatemala
Source: PLoS Negl Trop Dis. 2026 Sep 3;20(9):e0014654. doi: 10.1371/journal.pntd.0014654 (PMC13541171; doi:10.1371/journal.pntd.0014654)
Supplement: Supplementary file 7 [file pntd.0014654.s005.docx]

**Figure S1**. Where is the risk of acquiring dengue (multiple responses possible)
