# Supplementary material for: Preparing for the tiny predators: Community readiness and perceptions of copepod-based dengue control in eastern guatemala
Source: PLoS Negl Trop Dis. 2026 Sep 3;20(9):e0014654. doi: 10.1371/journal.pntd.0014654 (PMC13541171; doi:10.1371/journal.pntd.0014654)
Supplement: Peer review history

# Peer review: Decision Letter 0

**DOI:** 10.1371/journal.pntd.0014654.r001

**Authors:** Álvaro Acosta-Serrano, Rafael Maciel-de-Freitas

14 May 2026

Response to Reviewers Revised Manuscript with Track Changes Manuscript

Shaden Kamhawi

co-Editor-in-Chief

PLOS Neglected Tropical Diseases

orcid.org/0000-0003-4304-636XX

Paul Brindley

co-Editor-in-Chief

PLOS Neglected Tropical Diseases

orcid.org/0000-0003-1765-0002

**Additional Editor Comments:** **Journal Requirements:**

1) Please ensure that the CRediT author contributions listed for every co-author are completed accurately and in full.

At this stage, the following Authors/Authors require contributions: Valérie R Louis, Carlos Alberto Montenegro-Quinoñez, and Peter Dambach. Please ensure that the full contributions of each author are acknowledged in the "Add/Edit/Remove Authors" section of our submission form.

The list of CRediT author contributions may be found here: https://journals.plos.org/plosntds/s/authorship#loc-author-contributions

2) Some material included in your submission may be copyrighted. According to PLOS's copyright policy, authors who use figures or other material (e.g., graphics, clipart, maps) from another author or copyright holder must demonstrate or obtain permission to publish this material under the Creative Commons Attribution 4.0 International (CC BY 4.0) License used by PLOS journals. Please closely review the details of PLOS's copyright requirements here: <a data-cke-saved-href="https://journals.plos.org/plosntds/s/licenses-and-copyright" href="https://journals.plos.org/plosntds/s/licenses-and-copyright">PLOS Licenses and Copyright</a>. If you need to request permissions from a copyright holder, you may use the&nbsp;<a data-cke-saved-href="https://storage.googleapis.com/plos-published-prod/c4ff/Content%20Copyright%20Permission%20Form.pdf?X-Goog-Algorithm=GOOG4-RSA-SHA256&X-Goog-Credential=wombat-sa%40plos-prod.iam.gserviceaccount.com%2F20220817%2Fauto%2Fstorage%2Fgoog4_request&X-Goog-Date=20220817T210608Z&X-Goog-Expires=86400&X-Goog-SignedHeaders=host&X-Goog-Signature=cd9dc868773378f7033b9f8cfb91430167d6275df90bce01342a454f36512530b64f24736fbf68c4fd96da74d663b6ee95b3420bded8fb0e833af2390b896b8706643b476d7f532408921b60576d65b60617070900fd2bcd28eec4cd32fefcf007d3655df17fcc91023329a28479ce0d1c5758174eedcc9e3c1788c5f7dd8523f3ddd871e02c998a20f7dc78ec00f313d4b5a78ba2037a6c7ef0a05dc29817da9368065460c8d8446659f35f0458fefabd3101eb7f926e9cb7a0b53e7bbe47aaed3e0ba52f9af9c2b8f6d7d3a8871b205fc4cbe97384ed37c7604fd7eecfedccf49cba12820b2e3b649541cefde3103cd7d72731b3e8b0248439b4cba691ca8a" href="https://storage.googleapis.com/plos-published-prod/c4ff/Content%20Copyright%20Permission%20Form.pdf?X-Goog-Algorithm=GOOG4-RSA-SHA256&X-Goog-Credential=wombat-sa%40plos-prod.iam.gserviceaccount.com%2F20220817%2Fauto%2Fstorage%2Fgoog4_request&X-Goog-Date=20220817T210608Z&X-Goog-Expires=86400&X-Goog-SignedHeaders=host&X-Goog-Signature=cd9dc868773378f7033b9f8cfb91430167d6275df90bce01342a454f36512530b64f24736fbf68c4fd96da74d663b6ee95b3420bded8fb0e833af2390b896b8706643b476d7f532408921b60576d65b60617070900fd2bcd28eec4cd32fefcf007d3655df17fcc91023329a28479ce0d1c5758174eedcc9e3c1788c5f7dd8523f3ddd871e02c998a20f7dc78ec00f313d4b5a78ba2037a6c7ef0a05dc29817da9368065460c8d8446659f35f0458fefabd3101eb7f926e9cb7a0b53e7bbe47aaed3e0ba52f9af9c2b8f6d7d3a8871b205fc4cbe97384ed37c7604fd7eecfedccf49cba12820b2e3b649541cefde3103cd7d72731b3e8b0248439b4cba691ca8a">PLOS Content Copyright Permission&nbsp;Form</a>

Please respond directly to this email and provide any known details concerning your material's license terms and permissions required for reuse, even if you have not yet obtained copyright permissions or are unsure of your material's copyright compatibility. Once you have responded and addressed all other outstanding technical requirements, you may resubmit your manuscript within Editorial Manager.

Potential Copyright Issues:

i) Figure Figure 1.. Please (a) provide a direct link to the base layer of the map (i.e., the country or region border shape) and ensure this is also included in the figure legend; and (b) provide a link to the terms of use / license information for the base layer image or shapefile. We cannot publish proprietary or copyrighted maps (e.g. Google Maps, Mapquest) and the terms of use for your map base layer must be compatible with our CC BY 4.0 license.

Note: if you created the map in a software program like R or ArcGIS, please locate and indicate the source of the basemap shapefile onto which data has been plotted.

If your map was obtained from a copyrighted source please amend the figure so that the base map used is from an openly available source. Alternatively, please provide explicit written permission from the copyright holder granting you the right to publish the material under our CC BY 4.0 license.

If you are unsure whether you can use a map or not, please do reach out and we will be able to help you. The following websites are good examples of where you can source open access or public domain maps:

* U.S. Geological Survey (USGS) - All maps are in the public domain. (http://www.usgs.gov)

* PlaniGlobe - All maps are published under a Creative Commons license so please cite u201cPlaniGlobe, http://www.planiglobe.com, CC BY 2.0u201d in the image credit after the caption. (http://www.planiglobe.com/?lang=enl)

* Natural Earth - All maps are public domain. (http://www.naturalearthdata.com/about/terms-of-use/).

3) In the online submission form, you indicated that "The data can be received upon request through the corresponding author.". All PLOS journals now require all data underlying the findings described in their manuscript to be freely available to other researchers, either

1. In a public repository

2. Within the manuscript itself

3. Uploaded as supplementary information.

This policy applies to all data except where public deposition would breach compliance with the protocol approved by your research ethics board. If your data cannot be made publicly available for ethical or legal reasons (e.g., public availability would compromise patient privacy), please explain your reasons by return email and your exemption request will be escalated to the editor for approval. Your exemption request will be handled independently and will not hold up the peer review process, but will need to be resolved should your manuscript be accepted for publication. One of the Editorial team will then be in touch if there are any issues.

**Reviewers' comments:**

**Key Review Criteria Required for Acceptance?**

As you describe the new analyses required for acceptance, please consider the following:

**Methods**

-Are the objectives of the study clearly articulated with a clear testable hypothesis stated?

-Is the study design appropriate to address the stated objectives?

-Is the population clearly described and appropriate for the hypothesis being tested?

-Is the sample size sufficient to ensure adequate power to address the hypothesis being tested?

-Were correct statistical analysis used to support conclusions?

-Are there concerns about ethical or regulatory requirements being met?

Reviewer #1: Are the objectives of the study clearly articulated with a clear testable hypothesis stated?

Yes. The objectives are clearly articulated.

Is the study design appropriate to address the stated objectives?

Yes, the cross-sectional Knowledge, Attitudes, and Practices (KAP) survey design is appropriate for the research question.

Is the population clearly described and appropriate for the hypothesis being tested?

Yes. However, it is better to provide more details on the dengue burden of the selected community.

Is the sample size sufficient to ensure adequate power to address the hypothesis being tested?

The sample size of N = 346 is generally considered sufficient for a cross-sectional study to obtain reliable estimates of prevalence for key outcomes.

Were correct statistical analysis used to support conclusions?

1.) The methodology for calculating the compound score is confusing and needs more clarification for the reader.

2.) The use of stepwise regression to identify significant variables at the 0.1 level before running the final models is a common practice but is generally discouraged in modern epidemiological literature due to risks of inflated Type I error rates, selection bias, and difficulty in interpreting p-values.

3.) The authors state, "Missing data were excluded resulting in slightly varying denominators." This suggests a complete-case analysis. Please provide more details on missing data.

4.) The authors correctly note the use of Poisson regression for count data and state that the mean was "roughly equal to the variance," which is the core assumption of the Poisson model. Please confirm that the final models did not exhibit overdispersion, especially when running the models clustered at the community level.

5.) The authors mentioned that "The regressions were run with clustering at the community level using

the standard error command, “vce(cluster ‘community’)". Please provide more details on the purpose of using this function.

Are there concerns about ethical or regulatory requirements being met?

Please indicate if the ethical approval was obtained from the institution where data was analyzed.

Reviewer #2: Sample size calculation:

- The authors calculated 384 for simple random sampling, then used clustered sampling and reduced the sample size to 340. In this case shouldn’t the design effect make it larger? What I suggest to the authors is to, instead of explaining the drop from 384 to 340 (i.e., “logistical constraints… final required sample size of 340… deemed sufficient”), acknowledge that a design effect wasn’t explicitly used, and explain that the sample is primarily intended for descriptive and exploratory purposes rather than highly precise inferential estimates; and that 340 still gives roughly a 5–6% margin of error at 95% confidence for proportions around 50%.

Survey population:

- Can the authors clarify how the team ensured even coverage in relation to how the households were enumerated?

- The authors mentioned that systematic random sampling was used but fail to mention that a random starting position was chosen. I suggest that this be explicitly mentioned.

Questionnaire and Data collection

- The authors make mentions of creating the questionnaire “based on a thorough review of existing literature, including validated KAP survey”. Please cite and reference here the validated KAP surveys that this KAP survey was built upon.

- Please clarify how was the questionnaire translated into Spanish. Who translated the survey and was it back-translated to ensure validity?

Analysis

- Please mention how descriptive statistics were presented (i.e., in frequencies and percentages).

- Regarding ‘correct response’ definition – it may be clearer to specify that correctness was based on expert-defined criteria or pre-specified answer keys, rather than to say, ‘depending on the context’.

- Why was stepwise regression utilized? Please provide a short justification.

- What factors were model selection based on for logistic regression models (i.e., c statistic, AIC, etc)?

- What was the outcome estimate that the logistic regression models produced? Was it an adjusted odds ratio? Kindly specify.

Ethics and consent

- Kindly explain in how “confidentiality was strictly maintained”.

**********

**Results**

-Does the analysis presented match the analysis plan?

-Are the results clearly and completely presented?

-Are the figures (Tables, Images) of sufficient quality for clarity?

Reviewer #1: Does the analysis presented match the analysis plan?

As noted in the prior review of the Analysis section, the precise composition and maximum score of the compound score are still unclear in the results text (it mentions a score, but the denominator is not stated in the table).

Are the results clearly and completely presented?

1.) The regression table (Table 2) uses the score names (e.g., K- Dengue Transmission Routes (B2)) but does not explicitly state the maximum score for each outcome (K, P, A scores), nor does it state the total possible range or the mean/median score to provide context for the estimated coefficients. The authors should ensure this information is either in the table caption or the main text.

2.) The regression results are reported as Estimated Coefficients (Est.) and their 95% Confidence Intervals (CI). Since the models used are Poisson (for scores) and/or Logistic (for binary outcomes, though none of the outcome headers look like a binary variable), the Est. represents the log-rate ratio (Poisson) or log-odds ratio (Logistic). The text should clearly state what the estimates represent. The current text only describes the directionality.

Are the figures (Tables, Images) of sufficient quality for clarity?

Needs improvement.

In Table 2, the reference categories for Education, Water Access, Water Storage, Sanitation, and Perceived Dengue Risk are implied (the one without coefficients). For example, for "Education," the reference is "Up to Primary school." This should be explicitly stated in the table to aid reviewer and reader interpretation.

Reviewer #2: Descriptive / sample characteristics

- Consider moving “a likely consequence of daytime data collection when women were more likely to be present at home” to discussion section. Please present results without explaining them. Use the discussion section to explain findings.

- “Educational attainment was low overall” compared to…? Is there a ‘standard educational attainment’?

- In Table 1. the second to last column (%) should not consistently represent 100%. For example, the total number of “primary school education” should be 151/341 which is not 100%, rather it is 44.3%.

- What does the p value represent in Table 1. The table should only represent descriptive statistics not inferential statistics.

Household infrastructure and environmental risk factors

- Consider moving the sentence “This variability may impact household water storage practices and, consequently, mosquito breeding potential” to discussion. Do not explain findings in results section.

- Can the authors specify in the methods section how data on the types of water supply were obtained? It seems this information obtained in the KAP survey - If yes, this must be explicitly stated in methods.

- Consider moving Table S1 to the main body of the manuscript.

Knowledge, attitude and practice scores

- Consider rephrasing the following “While neither sex nor home ownership were not significantly associated with score outcomes,…”- the sentence has many negatives, and hard to interpret.

- “higher education demonstrated better knowledge” – Does this mean that higher education was associated with higher knowledge scores? What does ‘better knowledge’ mean?

Attitudes

- Please specify in the methods section how data on ‘perceptions’ was collected. Was a Likert scale used? What methodology and theory was used to inform this data collection process?

- Figure 4 caption is incomplete.

Regression

- “The variables sex, house ownership, house outdoor area and energy source were initially considered in the analysis but were not inlcuded in the final models because they were not statistically significant” – Please include all statistical analyses conducted in the supplemental files.

- Please specify what outcome was used in the regression analyses? Was it an ordinal variable including the min to max scores in each knowledge category?

- In Table 2, use a symbol to represent the reference category.

**********

**Conclusions**

-Are the conclusions supported by the data presented?

-Are the limitations of analysis clearly described?

-Do the authors discuss how these data can be helpful to advance our understanding of the topic under study?

-Is public health relevance addressed?

Reviewer #1: The conclusions sufficiently summarize the key findings on community readiness and acceptance for copepod-based control, which is the primary objective of this KAP study. The statement that interventions "could become a valuable addition" is appropriately cautious, given the study’s focus is on social feasibility, not entomological effectiveness.

However, for clarity, the authors must emphasize in the conclusion that community acceptance does not equate to effectiveness. While the high willingness signals a crucial public health opportunity, this must be followed by rigorous field trials to test the intervention's success in terms of larval reduction and safety within the specific environmental conditions and household storage practices observed in Eastern Guatemala. This distinction is vital for setting accurate expectations for policy makers and future research.

Reviewer #2: Discussion/conclusions

- Methodological strengths and limitations: Need to address limitations in recruitment methods used. Make clear that results must be interpreted with methodological caveats in mind (i.e., more women than men in the sample – what does this mean for interpretation?). Does your sample fully represent Guatemala’s urban and rural socioeconomic and demographic diversity?

- What about recall and social desirability bias due to the KAP survey method used?

- Any residual and unmeasured confounding from your statistical analyses?

**********

**Editorial and Data Presentation Modifications?**

Use this section for editorial suggestions as well as relatively minor modifications of existing data that would enhance clarity. If the only modifications needed are minor and/or editorial, you may wish to recommend “Minor Revision” or “Accept”.

Reviewer #1: (No Response)

Reviewer #2: No modifications

**********

**Summary and General Comments**

Use this section to provide overall comments, discuss strengths/weaknesses of the study, novelty, significance, general execution and scholarship. You may also include additional comments for the author, including concerns about dual publication, research ethics, or publication ethics. If requesting major revision, please articulate the new experiments that are needed.

Reviewer #1: Please see my comments above.

Reviewer #2: This manuscript examines knowledge, attitudes, and practices related to dengue prevention and biological control in Guatemala, addressing a timely and relevant public health issue. The study objectives are clearly articulated, and the cross-sectional KAP design is generally appropriate for the stated aims. Data collection across both urban and rural communities and the inclusion of composite KAP scores strengthen the potential utility of the findings for local public health programming.

However, several methodological and reporting limitations reduce the rigor and interpretability of the results. The sample size calculation is based on simple random sampling, yet a clustered sampling design was used without accounting for a design effect, raising concerns about precision and inferential validity. The household sampling strategy lacks sufficient detail (e.g., random start), and representativeness issues such as the overrepresentation of women are not adequately addressed. Questionnaire development and translation procedures require clearer documentation, including citation of validated KAP instruments and description of translation and back-translation methods. There are some minor grammatical errors that must be addressed. Additional information in the supplementary files needed.

The statistical analysis would benefit from stronger justification, particularly regarding the use of stepwise regression, model selection criteria, and clearer specification of regression outcomes. Results presentation occasionally conflates findings with interpretation, and several tables and figures require correction or clarification.

Overall, while the study has public health relevance and contextual novelty, some revisions are needed to improve methodological transparency, analytical rigor, and clarity before the findings can be reliably interpreted.

**********

PLOS authors have the option to publish the peer review history of their article (what does this mean?). If published, this will include your full peer review and any attached files.

If you choose “no”, your identity will remain anonymous but your review may still be made public.

**Do you want your identity to be public for this peer review?**  For information about this choice, including consent withdrawal, please see our Privacy Policy.

Reviewer #1: No

Reviewer #2: No

**Figure resubmission:**

**Reproducibility:**

To enhance the reproducibility of your results, we recommend that authors of applicable studies deposit laboratory protocols in protocols.io, where a protocol can be assigned its own identifier (DOI) such that it can be cited independently in the future. Additionally, PLOS ONE offers an option to publish peer-reviewed clinical study protocols. Read more information on sharing protocols at https://plos.org/protocols?utm_medium=editorial-email&utm_source=authorletters&utm_campaign=protocols

# Author comment: Author response to Decision Letter 1

**DOI:** 10.1371/journal.pntd.0014654.r002

2 Jul 2026

Attachment Submitted filename: Response to Reviewers.docx

# Editor report: Decision Letter 1

**DOI:** 10.1371/journal.pntd.0014654.r003

**Authors:** Álvaro Acosta-Serrano, Rafael Maciel-de-Freitas

6 Aug 2026

Dear Prof. Dambach,

We are pleased to inform you that your manuscript 'Preparing for the Tiny Predators: Community Readiness and Perceptions of Copepod-Based Dengue Control in Eastern Guatemala' has been provisionally accepted for publication in PLOS Neglected Tropical Diseases.

Before your manuscript can be formally accepted you will need to complete some formatting changes, which you will receive in a follow up email. A member of our team will be in touch with a set of requests.

Please note that your manuscript will not be scheduled for publication until you have made the required changes, so a swift response is appreciated.

IMPORTANT: The editorial review process is now complete. PLOS will only permit corrections to spelling, formatting or significant scientific errors from this point onwards. Requests for major changes, or any which affect the scientific understanding of your work, will cause delays to the publication date of your manuscript.

Should you, your institution's press office or the journal office choose to press release your paper, you will automatically be opted out of early publication. We ask that you notify us now if you or your institution is planning to press release the article. All press must be co-ordinated with PLOS.

Thank you again for supporting Open Access publishing; we are looking forward to publishing your work in PLOS Neglected Tropical Diseases.

Best regards,

Álvaro Acosta-Serrano

Section Editor

PLOS Neglected Tropical Diseases

Álvaro Acosta-Serrano

Section Editor

PLOS Neglected Tropical Diseases

Shaden Kamhawi

co-Editor-in-Chief

PLOS Neglected Tropical Diseases

orcid.org/0000-0003-4304-636XX

Paul Brindley

co-Editor-in-Chief

PLOS Neglected Tropical Diseases

orcid.org/0000-0003-1765-0002

***********************************************************

# Editor report: Acceptance letter

**DOI:** 10.1371/journal.pntd.0014654.r004

**Authors:** Álvaro Acosta-Serrano, Rafael Maciel-de-Freitas

Dear Prof. Dambach,

We are delighted to inform you that your manuscript, "Preparing for the Tiny Predators: Community Readiness and Perceptions of Copepod-Based Dengue Control in Eastern Guatemala," has been formally accepted for publication in PLOS Neglected Tropical Diseases.

We have now passed your article onto the PLOS Production Department who will complete the rest of the publication process. All authors will receive a confirmation email upon publication.

The corresponding author will soon be receiving a typeset proof for review, to ensure errors have not been introduced during production. Please review the PDF proof of your manuscript carefully, as this is the last chance to correct any scientific or type-setting errors. Please note that major changes, or those which affect the scientific understanding of the work, will likely cause delays to the publication date of your manuscript. Note: Proofs for Front Matter articles (Editorial, Viewpoint, Symposium, Review, etc...) are generated on a different schedule and may not be made available as quickly.

Soon after your final files are uploaded, the early version of your manuscript will be published online unless you opted out of this process. The date of the early version will be your article's publication date. The final article will be published to the same URL, and all versions of the paper will be accessible to readers.

For Research Articles, you will receive an invoice from PLOS for your publication fee after your manuscript has reached the completed accept phase. If you receive an email requesting payment before acceptance or for any other service, this may be a phishing scheme. Learn how to identify phishing emails and protect your accounts at https://explore.plos.org/phishing.

Thank you again for supporting open-access publishing; we are looking forward to publishing your work in PLOS Neglected Tropical Diseases.

Best regards,

Shaden Kamhawi

co-Editor-in-Chief

PLOS Neglected Tropical Diseases

Paul Brindley

co-Editor-in-Chief

PLOS Neglected Tropical Diseases
